# Common Ground Information Affects Reference Resolution: Evidence From Behavioral Data, ERPs, and Eye-Tracking

**DOI:** 10.3389/fpsyg.2020.565651

**Published:** 2020-11-30

**Authors:** Maria Richter, Mariella Paul, Barbara Höhle, Isabell Wartenburger

**Affiliations:** ^1^Cognitive Sciences, Department of Linguistics, University of Potsdam, Potsdam, Germany; ^2^Max Planck Institute for Human Cognitive and Brain Sciences, Leipzig, Germany; ^3^Berlin School of Mind and Brain, Humboldt-Universität Zu Berlin, Berlin, Germany; ^4^Psychology of Language Department, University of Göttingen, Göttingen, Germany

**Keywords:** perspective-taking, ERPs, eye-tracking, common ground, privileged ground

## Abstract

One of the most important social cognitive skills in humans is the ability to “put oneself in someone else’s shoes,” that is, to take another person’s perspective. In socially situated communication, perspective taking enables the listener to arrive at a meaningful interpretation of what is said (sentence meaning) and what is meant (speaker’s meaning) by the speaker. To successfully decode the speaker’s meaning, the listener has to take into account which information he/she and the speaker share in their common ground (CG). We here further investigated competing accounts about when and how CG information affects language comprehension by means of reaction time (RT) measures, accuracy data, event-related potentials (ERPs), and eye-tracking. Early integration accounts would predict that CG information is considered immediately and would hence not expect to find costs of CG integration. Late integration accounts would predict a rather late and effortful integration of CG information during the parsing process that might be reflected in integration or updating costs. Other accounts predict the simultaneous integration of privileged ground (PG) and CG perspectives. We used a computerized version of the referential communication game with object triplets of different sizes presented visually in CG or PG. In critical trials (i.e., conflict trials), CG information had to be integrated while privileged information had to be suppressed. Listeners mastered the integration of CG (response accuracy 99.8%). Yet, slower RTs, and enhanced late positivities in the ERPs showed that CG integration had its costs. Moreover, eye-tracking data indicated an early anticipation of referents in CG but an inability to suppress looks to the privileged competitor, resulting in later and longer looks to targets in those trials, in which CG information had to be considered. Our data therefore support accounts that foresee an early anticipation of referents to be in CG but a rather late and effortful integration if conflicting information has to be processed. We show that both perspectives, PG and CG, contribute to socially situated language processing and discuss the data with reference to theoretical accounts and recent findings on the use of CG information for reference resolution.

## Introduction

One of the most important social cognitive skills in humans is the ability to “put oneself in someone else’s shoes,” that is, to take another person’s perspective. In communication, perspective taking enables the listener to arrive at a meaningful interpretation of what is said (sentence meaning) and what is meant (speaker’s meaning) by the speaker (Grice, 1989). Beyond linguistic information, visual and other contextual information is taken into consideration incrementally (see, for instance, the *Coordinated Interplay Account* by Knoeferle and Crocker, 2006; see also Knoeferle and Crocker, 2007; Crocker et al., 2010; Münster and Knoeferle, 2017). Especially in reference processing, the listener may have to take the speaker’s perspective in order to decode the speaker’s communicative intention. A referent can be a person, an object, or a concept, to which the speaker refers with a so-called referring expression. Speakers can choose different forms of referring expressions (e.g., a full noun phrase, a pronoun etc.) in discourse to optimize information transfer. For instance, when a referent is first introduced in discourse, the speaker commonly selects an indefinite noun phrase (e.g., a woman enters the bar). In subsequent discourse, the speaker refers back to that referent with a definite noun phrase (e.g., the woman), a pronoun (e.g., she), or another definite description (e.g., the beautiful lady), which adds information to the referent (Schumacher, 2018). Although experimental research confirmed that the speaker mostly provides sufficient but no redundant information when using referring expressions, over- and under-informative utterances occur (e.g., Deutsch and Pechmann, 1982; Engelhardt et al., 2006; Davies and Katsos, 2010; Morisseau et al., 2013). In these cases, listeners may then face multiple possible referents within the linguistic and/or non-linguistic context. In order to understand which referent the speaker was referring to, the listener has to take the speaker’s perspective. This requires the calculation of mentally and/or perceptually shared information by both interlocutors, which is often called common ground (CG) information (e.g., Clark et al., 1983). With the present study we intend to better understand, if, when, and how information in privileged and CG is integrated during utterance processing.

A body of research has been concerned with this question. For a long time, existing parsing theories took two rather different, apparently contradictory views. On the one side there were theories that assume autonomous lexical and syntactic activation with contextual and other pragmatic constraints, such as CG, entering the parsing process only at a later stage at which the different sources of information are integrated (e.g., Ferreira and Clifton, 1986; Keysar et al., 2000; Epley et al., 2004b; Barr, 2008; Kronmüller et al., 2017). We will refer to these accounts as “*late integration*” accounts. On the other side, constraint-based theories assumed that all available information sources do immediately interact during the parsing process and guide the interpretation of a sentence (e.g., Altmann and Steedman, 1988; Spivey-Knowlton and Sedivy, 1995; Trueswell et al., 1999; Nadig and Sedivy, 2002; Hanna et al., 2003; Snedeker and Trueswell, 2003; Snedeker and Yuan, 2008). We will refer to these accounts as “*early integration*” accounts.

Data supporting the assumption of a late integration of pragmatic information during the parsing process stemmed from Keysar et al. (2000). They used a version of the referential communication game, also called director’s task (Glucksberg et al., 1975; Krauss and Glucksberg, 1977). In this game, objects are placed in a vertical array. A confederate (experimenter) sits on one side of the array and instructs an addressee (participant) on the other side of the array to manipulate the objects in a certain manner. Crucially, some of the objects are hidden from the experimenter’s view, giving the participant privileged access to them. In order to follow the experimenter’s instruction correctly, that is, to pick the correct referent, participants have to consider which objects are shared for both interlocutors (i.e., are in CG). In Keysar et al. (2000) the overt responses revealed that participants based their decision on CG information in most of the cases (around 80%), that is, they picked the objects in CG. However, the eye fixation data showed that the participants initially fixated the privileged object [i.e., the competitor that was exclusively visible for the participant, that is, in privileged ground (PG)] and only later turned their eyes to the object in CG (i.e., the target). This interference effect produced by the privileged object supports the view that CG does not immediately restrict the search for referents. CG information is rather integrated late with effort, after an initial egocentric interpretation might have even led to egocentric errors, that is, picking an object that is not in CG (*Egocentrism Account*). For the (limited) effects on cultural backgrounds on egocentric errors see Wu and Keysar (2007) and Wang et al. (2019).

Further evidence for late integration accounts was obtained by Barr (2008). He used a slightly different method to instantiate the CG vs. PG objects. Here, participants directed already more fixations to the CG objects before any verbal instruction was given to them. This indicated an anticipation that the confederate would refer to CG objects. However, after the verbal instruction (e.g., “click on the bucket”) the participants needed longer time to orient their gaze to the target object when an object with a label that constituted a phonological competitor (i.e., competitor condition, e.g., bucket-buckle) was present compared to a control condition without competitor. This held true for all competitors, independent of whether they were presented in CG or PG. Crucially, when comparing the effect of interference of competitors in CG vs. PG, no differences were revealed. This suggested that CG information did not attenuate the interference of competitors, as constraint-based theories would assume. These results were interpreted in the framework of the *Autonomous Activation Account*. It proposes that listeners initially actively attempt to take a speaker’s perspective in anticipation of a linguistic expression (i.e., in the phase before any verbal instruction is given). Then they fail to fully integrate CG information, because the lexical information given by the speaker autonomously activates the information in PG (i.e., the competitor).

In contrast to these two accounts, that considered CG integration as a rather late and effortful process in which egocentric errors may occur (Keysar et al., 2000; Barr, 2008; Wang et al., 2019), earlier and cognitively less demanding effects of perspective taking on reference resolution had also been found. This was the case in similar tasks when linguistic markers, such as color terms (e.g., red), (in)definite expressions (e.g., the/one of the), or scalar adjectives (e.g., big/small) were available to narrow down the relevant contrasts. For example, Hanna et al. (2003) used a version of the referential communication game in which a referring expression was either ambiguous with respect to two objects in CG or in which one of these objects was privileged. For instance, the confederate instructed listeners (participants) to “put the blue circle above the red triangle”. In conditions with two red triangles in CG, participants were equally likely to look at either. When one object was privileged, participants were more likely to look at the object in CG from the earliest moments and were faster to choose it, hence supporting an early integration. Also, Heller et al. (2008) presented displays which contained two pairs of size contrasting objects, for instance, a big duck (target) and a small duck (target-contrast), a big box (competitor) and a small box (competitor-contrast). There were two conditions: In the shared condition, all objects were in CG. In the privileged condition, one of the items belonging to a competitor-contrast (e.g., the small box) was in PG. Listeners received instructions with scalar adjectives, for example, “pick up the big duck”. The results showed that listeners immediately used the distinction between CG and PG. They thus integrated CG early, challenging a possible egocentric-first heuristic. This is consistent with other studies that found an early effect of CG information (e.g., Nadig and Sedivy, 2002; Hanna and Tanenhaus, 2004; Brown-Schmidt et al., 2008; Brown-Schmidt, 2012; Ferguson and Breheny, 2012). While the results of Heller et al. (2008) speak against an automatic egocentric bias in interpreting perspective-sensitive language, the authors do not claim a CG heuristic that directs attention only to mutual information. Instead they suggest that listeners are aware of the common or privileged status of information and use this distinction early in real-time reference resolution.

Other research has shown that the use of CG information, as well as a reduction of egocentric biases, is facilitated by rich discourse contexts such as when conversational context explicitly establishes what the confederate does and does not know through the use of questions (e.g., “What’s above the cow?”) (Brown-Schmidt et al., 2008). Similarly, active engagement in a task leads to earlier inferences about others’ perspectives, and boosts the immediate use of this information to anticipate others’ actions compared to passive observers (Ferguson et al., 2015). Finally, the motivation of participants plays a role: when there is a high motivation or incentive for integrating perspectives and when sufficient cognitive resources are available, participants can activate perspective taking abilities early on (Epley et al., 2004a; Cane et al., 2017). In sum, these findings indicate that CG information can be immediately processed, even involuntarily, and used early in the parsing process, contradicting late integration accounts.

Recent approaches have considered neurobiological data to disentangle early and late integration accounts of CG processing. From a neurobiological perspective the human brain enables rapid communication through a continually implemented perception-action cycle. That is, sensory input is perceived (e.g., the confederate’s speech), and generates a particular action (e.g., one’s own verbal response), which in turn results in a self-generated sensory input, and, again, in a certain response (Bornkessel-Schlesewsky and Schumacher, 2016). Crucially for the needs of CG integration, this perception-action cycle also allows for predictive coding, and, in case of a mismatch between prediction and input, instantiates an update and the modification of the internal model (Bornkessel-Schlesewsky and Schumacher, 2016). The neuronal implementations of these mechanisms have been investigated in recent years with event-related potentials (ERPs). A late positive ERP component (starting around 400–500 ms and lasting around 1000 ms post stimulus onset) was associated with reconceptualization or repair mechanisms (Schumacher et al., 2018), and with reference processing (Schumacher, 2009). In her neurocognitive model of reference resolution, Schumacher (2009) suggested that a late positive ERP component reflects additional processing costs that arise whenever a prior discourse representation has to be updated or modified (e.g., with the emergence of a new referent). Other ERP studies investigating referential aspects of language comprehension also revealed ERP effects such as the P600 (e.g., Osterhout and Mobley, 1995; van Berkum et al., 1999; Harris et al., 2000). Hoeks and Brouwer (2014) refer to the internal model during discourse comprehension as *Mental Representation of what is Communicated*, MRC. In their view, the P600 reflects the construction or revision of an MRC. If establishing reference turns out to be impossible, or leads to an implausible interpretation, a P600 will ensue, reflecting the reorganization of the MRC. A P600 may also appear in the absence of such serious problems, when a discourse entity needs to be accommodated, or when the referring expression needs some “pre-processing” before the antecedent can be successfully identified.

In addition to the P600 ERP effects there is evidence of another ERP component involved in referential processing. Referentially ambiguous nouns (e.g., “the girl” in a two-girl context) or pronouns (e.g., “David noticed John when he stood up.”) elicited a frontally dominant and sustained negative shift, called Nref effect (van Berkum et al., 1999; Nieuwland et al., 2007, for a review see van Berkum et al., 2007). Nieuwland et al. (2007) highlight that the frontal negative shift reflects genuine referential ambiguity in the current model of the discourse. Hoeks and Brouwer (2014) instead propose that each referring expression elicits an Nref response as soon as the search for an antecedent is instantiated.

Recently, Sikos et al. (2019) reported an Nref-effect as a marker of referential ambiguity in a perspective taking task. In their study, participants were asked to pick a referent from a display of four animals (e.g., “Click on the brontosaurus with the boots”) by a speaker who could only see three of the animals. A competitor (e.g., a brontosaurus with a purse) was either mutually visible, visible only to the listener, or absent from the display. Results showed that the mutually visible competitor elicited a referential ambiguity as reflected by an Nref-effect. Crucially, when listeners had privileged access to the competitor, the ERPs did not show evidence for a referential confusion–although participants were slower when the privileged competitor was present. The authors concluded that participants did not consider the competitor in PG to be a candidate for reference. This interpretation is in line with early integration accounts that allow a rapid integration of pragmatic information during online language comprehension and hence speak against late integration accounts. However, the finding is incompatible with “egocentric errors” in behavioral studies (e.g., Keysar et al., 2000), in which participants apply an egocentric interpretation strategy and choose the competitor in PG as the target item–and hence obviously consider it as a potential candidate for reference. Furthermore the finding is incompatible with interference effects from objects in PG that have been shown in a series of experiments by Barr (2008). Note, however, that the discussed studies also differ in the actual task design applied.

Barr (2016) called for the need to focus on the underlying processes and the use of joint data analysis routines. In the same year, Heller et al. (2016) aimed at solving the above mentioned traditional contradictions of early and late integration accounts by implementing the data of the original eye-tracking studies of Keysar et al. (2000) and Heller et al. (2008) in a Bayesian model of reference resolution. The model suggests that referring expressions are not interpreted relative to the CG or to one’s egocentric knowledge, but rather reflect the *Simultaneous Integration* of the two perspectives. In their probabilistic model, both the egocentric and the CG perspective are active in their referential domains (the referential domain is a contextually restricted set which is inferred and updated according to the current situation; here an egocentric domain and a CG domain is implemented). To gain information about the target referent, listeners simultaneously weigh evidence from both perspectives (Heller et al., 2016).

To disentangle the predictions of early and late integration accounts we here further investigated how listeners integrate egocentric and CG perspectives by adapting the well-established referential communication game of Keysar et al. (2000) to a computerized version. While we collected reaction time (RT) and accuracy data, we applied eye-tracking as well as electroencephalography (EEG) to study the timing and the underlying mechanisms of CG integration. Both methods, eye-tracking and EEG, offer a very high temporal resolution. They are therefore especially suitable to explore the temporal dynamics of the integration of CG information. Importantly, while eye movements might be affected by attentional processes that are unrelated to referent identification, EEG might be better suited to gain knowledge about the functionally distinct processes that underpin perspective taking. Our first study (ERP, Experiment 1) thus offers the opportunity to disentangle different aspects of the comprehension of referential expressions. In addition, EEG allows to draw inferences about the underlying neural mechanisms of CG integration and can be directly compared to the findings of Sikos et al. (2019). Our second study (eye-tracking, Experiment 2) with a mostly identical design to the ERP-Experiment allows for a descriptive alignment of eye-tracking results with our ERP data and provides further insights into the interaction of language comprehension and the perception of the visual world (for a recent short methodological overview see Rodriguez Ronderos et al., 2018). In addition to the ERP analysis, we appended an exploratory time-frequency analysis (TFA) of the EEG data in the Supplementary Material, which might provide insights about the mechanisms underlying CG processing. At the behavioral level, Experiment 2 can be taken as an attempt to replicate Experiment 1.

The *Simultaneous Integration Account* would predict that both the egocentric and the CG perspective are active when engaged in referential communication. However, depending on the evidence triggered by the specific task or the array, either egocentric or CG behavior may be enforced (Heller et al., 2016). Since our design was very similar to that of Keysar et al. (2000), egocentric behavior may guide at least initially the perspective taking behavior. This would lead to a rather late, and effortful integration of CG information during the parsing process. If participants first consider the object in PG to be the target and then switch to the (correct) target object in CG in a competitor (here: conflict) condition, some kind of discourse updating or reconceptualization has to take place. According to previous ERP studies in the field (see above), this late and effortful integration of CG would elicit a late positivity in the ERPs. This expectation is therefore in contrast to the findings of Sikos et al. (2019) who argue that the object in PG is not considered to be a potential referent in the display and therefore would not elicit a specific ERP response (in their case an Nref component). For the behavioral and eye-tracking data, we expect to replicate the findings of Keysar et al. (2000). That is, we expect more errors and/or longer RTs in the conflict condition in which participants probably have to suppress their egocentric bias. Accordingly, eye-tracking should reveal earlier looks to the competitor in PG, and later looks to the target in CG in the conflict condition in comparison to a condition without conflict. On the other hand, if the clear instruction, the integrated practice phase, and the high repetition rate in our experimental design promotes the CG perspective taking behavior, CG information would be considered immediately. In this case we would not expect discourse updating and thus no late positivity in the ERPs. Rather, we would expect no effects in ERP signatures as a result of CG integration.

## Materials and Methods

### Experiment 1: EEG

#### Participants

Thirty-six students of the University of Potsdam (17 female, *M* 24.6 years, age range 20–31 years) participated in the study. All participants were native German speakers, reported normal or corrected-to-normal vision, normal hearing, no neurological problems, and were right-handed as assessed by a German version of the Edinburgh Handedness Inventory (Oldfield, 1971). Nine participants were excluded for further ERP analysis due to technical problems during the recording (*n* = 2), or because less than 50% artifact-free trials survived the artifact rejection procedure in the critical conditions (*n* = 7). Thus, 27 participants entered the final ERP analysis (12 female, *M* = 24.8 years, age range 20–31 years). All participants gave written informed consent according to the local Ethics Committee of the University of Potsdam. Participants received course credits or financial compensation for their participation.

#### Materials and Design

**FIGURE 1:**
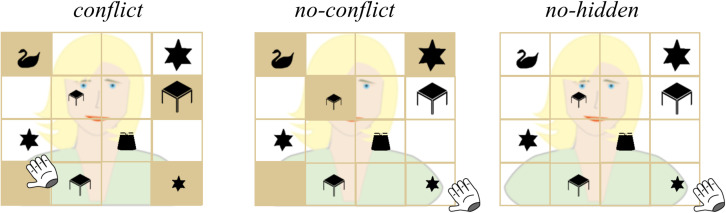
Examples of the experimental display for the conditions *conflict*, *no-conflict*, and *no-hidden* for the request “Move the small star to the top!”. The target in each condition is indicated by the position of the hand cursor for visualization purposes only. In the *conflict* condition, the smallest star at display was privileged, i.e., occluded from the avatar’s view. Therefore the medium-sized star, which was the smallest star in CG, formed the target. In the *no-conflict* condition, the object that fit the avatar’s request best (i.e., the smallest star) was in CG, thus not occluded from the avatar’s view, therefore no conflict arose. In the *no-hidden* condition, all objects were in CG. In the *filler* condition (not analyzed, not displayed), the avatar would ask for the CG single object (e.g., skirt in the display to the left).

Participants played a computerized version of the referential communication game (see Keysar et al., 2000). In this game, a virtual 4 × 4 grid was presented on a computer screen. Each display of the grid contained two object triplets with three differently sized objects (i.e., small, medium, big), two single objects (distractors), and eight empty slots (see Figure 1). We used two object triplets to prevent that participants would know, after a few trials, from the beginning of the display, which objects could potentially become the target. Thirty-two different objects were used to build the object triplets, and 18 other objects were used as single objects. All objects were black-and-white drawings representing man-made concrete objects like clothes, furniture or vehicles [e.g., Rock (skirt), Tisch (desk), or Zug (train)] or natural entities like celestial bodies, fruits, or animals [e.g., Stern (star), Apfel (apple), or Frosch (frog)]. The distribution of objects in the 16 slots of the 4 × 4 grid was fully randomized and changed across trials and participants. The virtual confederate (henceforth termed avatar) was displayed behind the grid and provided auditory instructions of the form: “[Move the] [target size] [target object] [to the top]!” (e.g., “[Move the] [big/small] [star/apple/frog/…] [to the top]!”; German: “[Bewege den] [großen/kleinen] [Stern/Apfel/Frosch…] [nach oben]!”). Notably, in German, both the determiner and the adjective are marked for gender. Therefore all nouns used were masculine, indicated by the gender-marked accusative direct determiner “den” (the), and the adjective-suffix “–en”. Due to this, a possible disambiguation before the onset of the noun was avoided. The instructions were pre-recorded by a trained native German female speaker and presented phrase by phrase with a fixed timing (_0__ms_[Bewege den] _1000__ms_[kleinen/großen] _1650__ms_[target object] _2950__ms_[nach oben]) (_0__ms_[Move the] _1000__ms_[small/big] _1650__ms_[target object] _2950__ms_[to the top]). Accordingly, the critical noun phrase (i.e., the [target object]) always started 1650 ms after the beginning of the auditory onset of the sentence (i.e., the [Bewege den]). The mean length of the nouns was 785 ms (±135 ms). Nevertheless, the overall sentences sounded prosodically well-formed as the phrases were cut out of natural recordings of the full sentences that were spoken in a relatively slow speech rate by the trained speaker. Participants had to select the target object via mouse click and had to drag and drop it on a field above the virtual grid. Dragging and dropping of the target objects were self-paced, meaning that participants were free to click on the object as soon as they had made their decision.

The crucial feature of the referential communication game is the manipulation of visual access to certain objects in the grid from the perspectives of the avatar and the participant, respectively. In three out of four conditions (conflict, no-conflict, filler), four slots in the grid contained a backboard that occluded their content from the avatar’s view. In the fourth condition, no-hidden, all slots were in CG. The no-hidden condition served as a control for effects of the mere presence of occluded slots in the grid (i.e., if there would be no differences between the no-hidden and the no-conflict condition, we could conclude, that the mere presence of occluded slots in the grid did not induce some unspecific computation of ground or did not affect general attentional processes). Positions of the backboards randomly changed from trial to trial. Three out of four of these occluded slots contained objects: two contained one object of the two object triplets, and one slot contained one of the two single objects (distractor). One of the four slots was empty. Since the participant had privileged access to the objects in these slots, we term them privileged objects (PG objects). For all other objects in the grid, both the avatar and the participants had visual access. They were in CG, and are henceforth termed CG objects. We created four different conditions: in *conflict trials*, one of the privileged objects fit the avatar’s request best from the perspective of the participant (e.g., the small star). In this condition the smallest star was a privileged object. Participants then had to consider which objects were visually shared, thus in CG, to select the correct object (“target”; e.g., the medium-sized star). In *no-conflict trials*, the object that fit the avatar’s request best from the perspective of the participant was in CG. In *no-hidden trials*, there were no occlusions at all and therefore all objects were in CG (please see Figure 1 for a detailed example of experimental displays). In *filler trials*, the target object was one of the two single objects (distractors) that was in CG (not at display in Figure 1). In all experimental conditions, the onset of the noun (i.e., [target object]) marked the point of disambiguation. In total, the EEG experiment consisted of 256 trials, 64 per condition (*conflict*, *no-conflict*, *no-hidden*, *filler*). Filler trials were not further analyzed. The distribution of conditions throughout the experiment was fully randomized and changed across participants.

#### Procedure

Participants were seated approximately 70 cm in front of the computer screen. A computer mouse was placed at a comfortable distance on a desk in front of the screen. RTs and accuracy measures were obtained via mouse click. All participants used their dominant right hand to navigate the computer mouse. In advance of the experimental phase, participants were instructed to mind the avatar’s perspective, which was supported by rotating the grid and showing the avatar’s view on the grid. This demonstrated to the participants that the avatar was not able to see the objects that were in slots with a wooden background. In addition, participants underwent a practice phase with nine practice trials during which they received corrective feedback (two-step instruction similar to Wang et al., 2016). For instance, participants received the instruction “[Move the] [small star] [to the top]!”. If they then chose the privileged object that is the smallest star at display in a *conflict* trial, the feedback they received from the avatar was: “Oh, I didn’t see that star. I meant the other small star!”.

Every trial started with a fixation cross, which was presented in the center of the screen for 1000 ms. Then, the empty grid with occlusions in four varying positions (with the exception of the no-hidden condition) was filled with the objects for 750 ms. Participants had time to view the grid for 500 ms. Then the avatar gave the auditory instruction, which was provided via headphones. Once the participants had made their choice, they clicked on the target with the computer mouse and dragged the object to a rectangle placed above the grid on the computer screen. Then, the next trial started.

During the test phase, the EEG was recorded and RTs and accuracy rates were measured. The RTs were measured for the first click on the target object starting from noun onset. The stimulus presentation and randomization was controlled by Presentation^®^ software version 18.1 (Neurobehavioral Systems). After the experiment participants were asked to fill out a debrief form about their intuitions concerning the purpose of the study and the strategies they used.

#### EEG Recordings

The EEG was recorded with a 32-channel active electrode system (Brain Products^®^, Gilching, Germany). 27 electrodes were placed on the scalp within an elastic soft cap (EASY CAP^®^, Inning, Germany) according to the 10/20 system (American Clinical Neurophysiology Society, 2006) at the following scalp positions: F7/8, F5/6, F3/4, FC3/4, C5/6, C3/4, CP3/4, P3/4, P7/8, PO3/4, AFz, Fz, FCz, Cz, CPz, Pz, and POz. The ground electrode was placed at FP1. The electrooculogram (EOG) was recorded with four additional electrodes. To detect blinks and vertical eye movements (vertical EOG), one electrode was placed above and one electrode below the participant’s right eye. To detect horizontal eye movements (horizontal EOG), electrodes were placed at the outer canthi of the left and the right eye. Impedances were kept below 5 kOhm. The EEG data was recorded with a sampling rate of 1000 Hz. The left mastoid served as the online reference electrode, but the recording was re-referenced offline to the averaged signal of the left and right mastoids.

#### Behavioral Data Analysis

The behavioral data comprised accuracy and RT measures. For the accuracy data, correct and incorrect responses were counted for each participant per condition (conflict, no-conflict, no-hidden). The total number of correct responses per condition was then transformed into percentage values to determine the accuracy rate. The accuracy rate for each condition and participant was then averaged across participants (*n* = 27). RTs were measured in ms relative the onset of the critical noun. Prior to the analysis, RTs with negative values (i.e., reactions before the onset of the noun), wrong responses, and “double clicks” on the target were removed using MS Excel^®^ (Version 2010). The remaining RTs were averaged for each condition per participant and then averaged across participants. To detect differences in behavior in relation to the three experimental conditions, an ANOVA with Condition (three levels: conflict, no-conflict, no-hidden) as within-subjects factor was run for both accuracy and RT measures separately. Whenever the main effect of condition reached significance (*p* < 0.05), *post-hoc* paired-samples *t*-tests controlled for multiple comparisons (Bonferroni corrected *p* = 0.017) were calculated. This was done to further examine the differences between conditions. Descriptive statistics as well as ANOVAs were carried out with the statistics software IBM^®^ SPSS Statistics for Windows (Version 23.0).

#### ERP Data Analysis

For ERP data preprocessing, the Brain Vision Analyzer software (version 2.0.2; Brain Products^®^, Gilching, Germany) was used. Raw data were filtered offline by applying a Butterworth zero-phase filter (low cutoff: 0.3 Hz; high cutoff: 70 Hz; slope: 12 dB/oct) to exclude slow signal drifts and muscle artifacts. In addition, a notch filter of 50 Hz was applied to remove line noise induced by electrical devices during testing. Artifacts caused by vertical and horizontal eye movements were corrected by the algorithm of Gratton et al. (1983). An automatic artifact rejection procedure was used to reject blinks, flat signals, and drifts in the time window of −200 to 1500 ms relative to the onset of the critical noun in the target sentence. The following criteria were set to automatically mark channels as bad: Maximal allowed voltage step: 20 μV/ms, maximal allowed difference of values: 75 μV per 150 ms time interval, minimal allowed amplitude: −75 μV, and a maximal allowed amplitude: 75 μV, and lowest allowed activity in intervals: 0.5 μV. Importantly, each trial was additionally examined visually and any remaining eye-blinks or eye movement artifacts were removed. Participants for whom less than 50% of trials in the noun onset time window survived the artifact rejection procedure were removed from further analysis (*n* = 7). Moreover, only trials in which participants selected the correct object (i.e., the target), entered the final analysis. In total, 23 trials (out of 6912 trials of the remaining 27 participants) with an incorrect response were removed (conflict condition: two trials, no-conflict condition: eight trials, no-hidden condition: 13 trials). Overall, the artifact rejection procedure and the deletion of incorrect trials resulted in a rejection of 21.55 ± 12.91% of trials (conflict condition: 22.05 ± 11.94%, no-conflict condition 21.12 ± 13.42%, no-hidden condition: 21.47 ± 13.38%). The amount of excluded trials did not differ across conditions as revealed by a repeated measures ANOVA with the factor condition as within subjects factor (*F*(3,78) = 1.23; *p* = 0.303, n*p*^2^ = 0.045).

For statistical analysis, we computed non-parametric cluster-based permutation analyses. This test calculates a cluster *t*-statistic that sums across temporally and spatially adjacent point-wise *t*-values that exceed a predefined threshold. This cluster *t*-statistic is then compared to a null-hypothesis distribution of cluster *t*-values that are generated via a Monte Carlo permutation approach. We used 1000 random permutations to generate a distribution of the null hypothesis with sufficient precision to control family wise error rate to α < 0.05, as suggested in Maris and Oostenveld (2007). The statistics was run two-tailed and within-subjects, with a minimum number of two significant (α < 0.05) electrodes to form a cluster. 50 ms running time windows were calculated, and considered as significant when they were significant over the entire time window. The cluster-based permutation analysis was performed with the open source software Fieldtrip for EEG/MEG analysis (Oostenveld et al., 2011) in MATLAB^®^ (2015b, MathWorks, Natick, MA, United States).

In addition to the ERP analysis, an exploratory TFA was performed on the EEG data. This was done since amplitude increases and decreases in specific frequency bands may provide further information about the underlying brain functions. Details regarding the analysis and full results are provided as Supplementary Materials.

### Experiment 2: Eye-Tracking

#### Participants

Twenty-nine native speakers of German (15 female; mean age: 24.3, range: 18–34) participated in the experiment. All of them gave written consent prior to the experiment, were naïve to the purpose of the study, and did not participate in Experiment 1. The participants received either course credits or financial compensation for their participation. All participants were right-handed as assessed by a German version of the Edinburgh Handedness Inventory (Oldfield, 1971), and had normal or corrected-to-normal vision. Two participants were excluded from the experimental cohort due to technical problems during the recordings, which resulted in an experimental breakup. Thus, 27 participants (14 female, mean age: 24.2, range 18–34) entered the final behavioral and eye-tracking analyses.

#### Materials and Design

Materials and design were almost identical to Experiment 1 (please refer to section “Materials and Design”). However, the amount of trials was reduced from 256 to 112 trials, with 28 trials per condition (*conflict*, *no-conflict*, *no-hidden*, *filler*). In addition, the position of the target and the privileged object in the grid was constrained so that these two objects could not appear in horizontally, vertically, or diagonally neighboring slots of the grid. This was done to minimize the misclassification of looks in the dense 16-slot-grid during data analysis. As in Experiment 1, filler trials were not further analyzed.

#### Procedure

The eye-tracking camera was attached at the middle of the lower edge of the PC monitor. The background screen color was set to dark gray. The participants were seated at a distance of 62–67 cm from a 22 inch (1680 × 1050 pixel) TFT PC monitor. The sitting position of the participants and the eye-tracking camera were adjusted checking that the pupils were recognized by the eye tracker in the center of a virtual box of the iViewRED-m application (SensoMotoric Instruments^®^, Teltow, Germany). The system was calibrated to the participants’ right eyes with a nine-point automatic calibration. For the calibration, a black dot was presented at different positions on a light gray background. In case of suboptimal calibration results, the procedure was repeated until the spatial precision of the gaze was classified as adequate by the system and by the experimenter.

Identical to Experiment 1, after calibration, a clear introduction was given by the avatar. A practice phase followed with a two-step corrective feedback (for details please see section “Procedure”). Then the experimental phase started with the presentation of four blocks á 28 trials. The task was the same as in Experiment 1. Participants used their right hand to navigate a computer mouse in order to click on the target and drag the object to a rectangle placed above the grid on the computer screen. After each block, a short, self-paced pause was inserted. Since participants were allowed to minimally move during the pause, the calibration procedure was repeated in advance of each block. During the test phase, eye-gaze data was recorded and RTs and accuracy rates were measured. The RTs were measured for the first click on the target object starting from noun onset.

#### Eye-Tracking Recordings

Eye movements were recorded with an SMI RED-m Eye-Tracker (SensoMotoric Instruments^®^, Teltow, Germany). Only the participants’ right eyes were tracked using SMI’s “smart right binocular mode”. With this mode, the system tracks gaze data every 8.33 ms (sampling frequency 120 Hz) and offers a spatial accuracy of 0.5–1°. The recovery time after track loss lies at 250 ms.

#### Behavioral Data Analysis

The behavioral data of the eye-tracking cohort was analyzed in the same way as the behavioral data of the EEG cohort. Please refer to section “Behavioral Data Analysis” for details of the analysis.

#### Eye-Tracking Data Analysis

The eye-tracking analysis (preprocessing, statistics) was performed with the free statistics software R^®^ (R Core Team, 2015). Only trials with correct responses entered the final analysis (*M* = 99.67 ± 0.44%). Data points for which the eye tracker could not determine the gaze position were removed. The overall track loss was on average 4.3%. Since the objects could appear in each of the 16 slots of the vertical array, we created 16 equally sized (170 × 170 pixels) spatial areas of interest (AoI) corresponding to the slots in the array. All gaze positions were automatically classified as being in one of the 16 AoIs or not.

**FIGURE 2:**
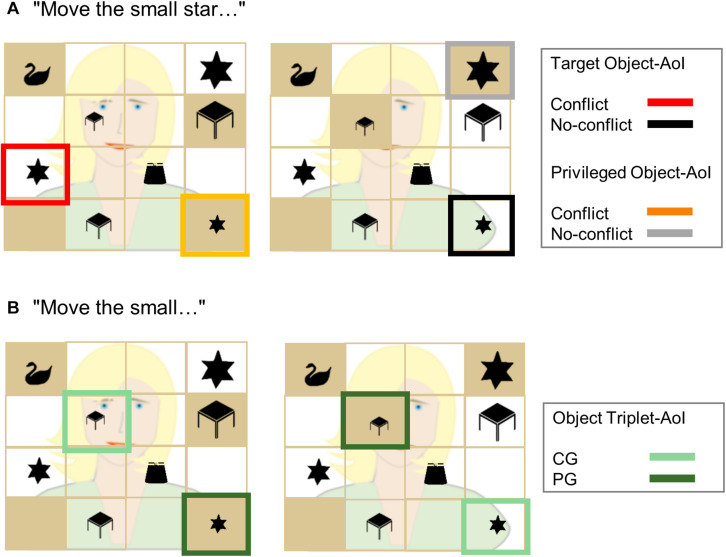
Example displays illustrating the three functional AoIs. In panel **(A)**, for the sentence “Move the small star…,” the target object-AoI for the conflict (red) and no-conflict (black) conditions, and the privileged object-AoI for the conflict (orange) and no-conflict (gray) conditions are illustrated. The analyses were run for the noun time window. In panel **(B)**, for the sentence “Move the small…,” the object triplet-AoI is depicted. All small objects of the two object triplets (here: small star, small desk) were assigned to the common ground (CG, light green) or the privileged ground (PG, dark green) condition according to their initial shared/privileged status. The analyses for the object triplet-AoI were carried out for the adjective time window, *before* listeners had the possibility to focus their attention on one of the triplets (e.g., the stars or the desks) or, in other words, *before* the trial could be assigned to a certain condition (i.e., conflict: left display, no-conflict: right display).

Next, we defined functional AoIs. The first functional AoI formed the area in which the target appeared. We will call this “target object”-AoI. For instance, given the instruction “Move the small star to the top!” the target would be the medium-sized star in the conflict condition and the small star in the no-conflict condition (for an illustration please refer to Figure 2A). The second functional AoI formed the area in which an object in PG appeared. We will call this “privileged object”-AoI. As an example, given the instruction “Move the small star to the top!” the privileged object would be the small star in the conflict condition and the big star in the no-conflict condition, both of which were hidden from the avatar’s view. Please note that only in the conflict condition, the object in PG provided a “real” competitor to the target object, since it had the potential to interfere with the target in CG (e.g., the medium sized star). In the no-conflict condition, however, participants were not expected to look at the object in PG (“the big star”). Still, it represents the object of comparison in PG when comparing the conflict and the no-conflict conditions (Figure 2A) (an alternative analysis of looks to the medium-sized object is presented as Supplementary Material). The third functional AoI incorporated all small or big objects of the two presented object triplets of the conflict and no-conflict condition trials (e.g., the small star and the small desk when the adjective was “small”; the big star and the big desk when the adjective was “big”; Figure 2B). Some of these objects were in CG and some of them were in PG.

Crucially, we created the “object triplet”-AoI to investigate early anticipatory looks to the objects in CG and PG, before the conditions (i.e., conflict, no-conflict) became evident for the participant. That is, the possibility to assign a sentence to one of the conditions only started with the presentation of the noun phrase, in dependency of the target item and the given occlusions. After the presentation of the noun, only one of the object triplets contained the target (e.g., the small star). However, in the preceding adjective time window, on which we focused in this analysis, participants could not know to which object triplet the target item belonged (e.g., the small star OR the small desk). We thus gave the third functional AoI the condition-neutral term “object triplet”-AoI (Figure 2B). We analyzed the looking behavior in the adjective time window comparing the looks to the objects in CG and PG of both object triplets. Crucially, if participants anticipated objects in CG to be the target, we should see more looks to the objects in CG compared to objects in PG in the adjective time window already.

The gaze data was first averaged across trials for each condition within participants, and then across participants for the grand average. Proportions of looks to the functional AoIs were calculated (values between one and zero).

Similar to the ERP analysis, we used the nonparametric cluster-based permutation analysis in order to detect reliable differences between conditions across time. Due to the strengths of nonparametric cluster-based permutation analyses (i.e., better control for multiple comparisons and the reduction of Type I errors) these analyses have also become more common in eye-tracking research (Holzen and Mani, 2012; Barr et al., 2014). For preprocessing and the statistical analysis we used the R package “eyetrackingR” (Dink and Ferguson, 2015). Non-AoI looks were treated as missing data. Then, we defined three time windows of interest: the adjective time window (1000–1650 ms post auditory onset), the noun time window (1650–2650 ms post auditory onset), and the post noun time window (2650–3650 ms post auditory onset). In each of these broader time windows, a time course based on 50 ms time bins was created, and proportion of looking times within each time-bin was summarized. Next, within the summarized time-bin data, adjacent time bins that passed the test-statistic threshold (α < 0.05, two-tailed *t*-test), were assigned into groups (clusters). This output was taken for the cluster-based permutation analysis (Maris and Oostenveld, 2007). This analysis took a summed statistic for each cluster, and compared it to the “null” distribution of sum statistics obtained by shuffling the data and extracting the largest cluster from each resample. Parallel to the ERP analysis, 1000 iterations were performed in the bootstrap resampling procedure.

## Results

### Experiment 1: EEG

#### Behavioral Findings

Accuracy was high for all participants (*n* = 27) across all conditions (*M* = 99.67 ± 0.40% accuracy rate). Errors occurred in all three conditions (accuracy conflict: *M* = 99.88 ± 0.42%; accuracy no-conflict: *M* = 99.54 ± 0.85%; accuracy no-hidden: *M* = 99.25 ± 1.40%). An ANOVA with Condition (3 levels: conflict, no-conflict, no-hidden) as within-subjects factor revealed no effect of condition (*F*(2,52) = 2.77; *p* = 0.09, n*p*^2^ = 0.096). However, perspective taking had its costs as revealed by RT measures: RTs measured relative to the onset of the critical noun (e.g., “star”) showed that participants were on average 184.22 ms slower in the conflict condition (*M* = 1469.07 ± 364.17 ms) compared to the means of the no-conflict (*M* = 1289.38 ± 375.18 ms) and no-hidden conditions (*M* = 1280.30 ± 367.80 ms). An ANOVA with Condition (three levels: conflict, no-conflict, no-hidden) as within-subjects factor revealed a significant effect of condition (*F*(2,52) = 31.52; *p* < 0.001; n*p*^2^ = 0.548). *Post-hoc* paired-samples *t*-tests controlled for multiple comparisons (Bonferroni corrected *p* = 0.017) revealed that RTs differed significantly between the conflict vs. no-conflict condition (*t*(26) = 6.47, *p* < 0.001), and between the conflict vs. no-hidden condition (*t*(26) = 7.49, *p* < 0.001) with conflict trials being longer than no-conflict and no-hidden trials. There was no significant difference between the no-conflict and the no-hidden conditions (*t*(26) = 0.33, *p* = 0.743).

#### ERP Results

**FIGURE 3:**
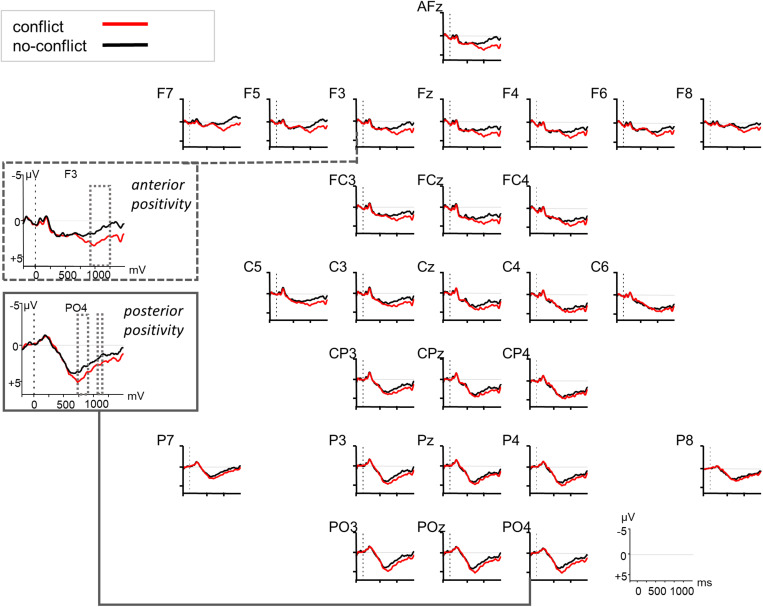
Grand averaged ERPs (*n* = 27) in response to the conflict (red) vs. no-conflict condition (black) relative to the onset of the critical noun (at 0 ms, dotted vertical line). F3 and PO4 are highlighted as example electrodes for the anterior and posterior positivity. The dotted squares indicate significant time windows as revealed by a cluster-based permutation test.

**FIGURE 4:**
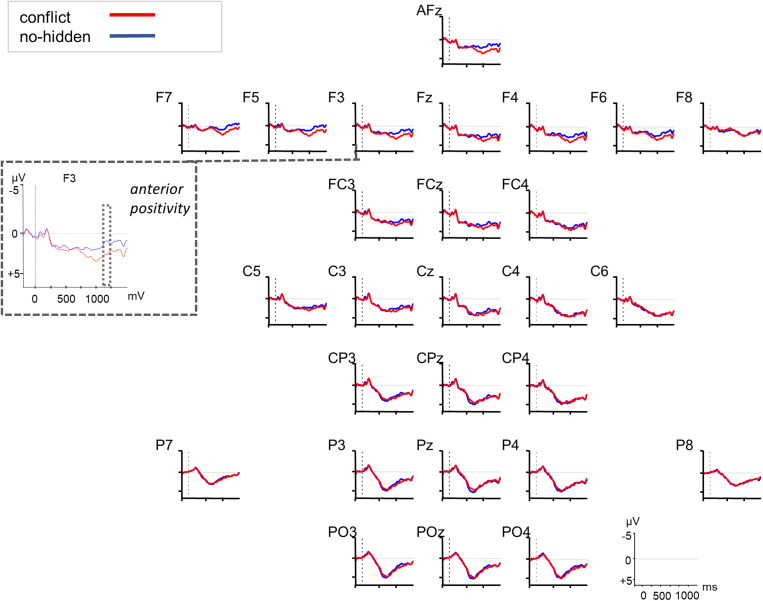
Grand averaged ERPs (*n* = 27) in response to the conflict (red) vs. no-hidden condition (blue) relative to the onset of the critical noun (at 0 ms, dotted vertical line). F3 is highlighted as example electrode for the anterior positivity. The dotted squares indicate the significant time window as revealed by a cluster-based permutation test.

**FIGURE 5:**
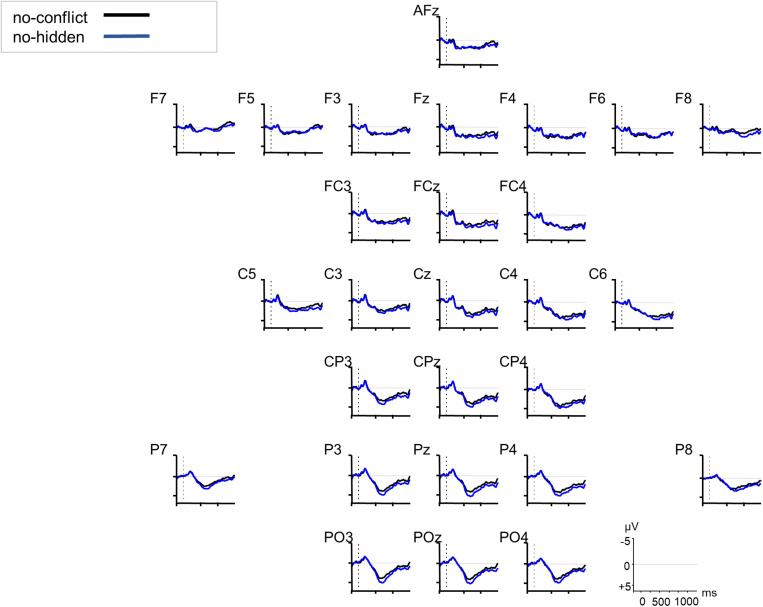
Grand averaged ERPs (*n* = 27) in response to the no-conflict (black) vs. no-hidden condition (blue) relative to the onset of the critical noun (at 0 ms, dotted vertical line). No significant time window was revealed by the cluster-based permutation testing.

The increased processing costs for the conflict condition as evidenced in the RT data were also reflected in the ERPs through modulations of a late positivity (see Figure 3). The cluster-based permutation analysis (Maris and Oostenveld, 2007) of the ERP data revealed two significant positive channel-time clusters for the comparison of the conflict vs. no-conflict condition over posterior brain areas 750–850 ms relative to noun onset (cluster *t*-statistic = 4031, *p* = 0.022), and over anterior and posterior brain areas 900–1250 ms relative to noun onset (cluster *t*-statistic = 31666, *p* = 0.001) (see Figure 3). The comparison of the conflict vs. no-hidden conditions (see Figure 4) showed a positive channel-time cluster over anterior brain regions only, 1100–1200 ms relative to noun onset (cluster *t*-statistic = 3624, *p* = 0.018). No significant channel-time clusters were revealed for the comparison of the no-conflict vs. no-hidden conditions (see Figure 5).

### Experiment 2: Eye-Tracking

#### Behavioral Findings

As for Experiment 1, the accuracy results of the eye-tracking cohort were high for all participants (*n* = 27) across all conditions (*M* = 99.67 ± 0.44%; conflict condition: *M* = 99.60 ± 1.14%; no-conflict: *M* = 99.21 ± 1.51%; no-hidden: *M* = 99.87 ± 0.69% accuracy rate). The ANOVA with condition (three levels: conflict, no-conflict, and no-hidden) as within-subjects factor revealed no significant effect of condition (*F*(2,52) = 1.97; *p* = 0.158, n*p*^2^ = 0.070) with respect to the accuracy rates. Thus, even in the conflict condition, participants mastered the integration of CG.

Similarly, the RTs relative to the onset of the critical noun (e.g., “star”) replicated those of the EEG cohort, with the ANOVA resulting in a significant effect of condition (*F*(2,52) = 27.38; *p* < 0.001, n*p*^2^ = 0.513). Participants were on average 202.30 ms slower in the conflict condition (*M* = 1570.20 ± 497.65 ms) compared to the means of the no-conflict (*M* = 1351.58 ± 526.68 ms) and no-hidden conditions (*M* = 1384.23 ± 489.60 ms). Paired-samples *t*-tests controlled for multiple comparisons (Bonferroni corrected *p* = 0.017) indicated that RTs differed significantly for the comparison of the conflict vs. no-conflict condition (*t*(26) = 7.09, *p* < 0.001), and for the conflict vs. no-hidden condition (*t*(26) = 5.41, *p* < 0.001) with RTs in the conflict condition being longer than in the other two conditions. There was no significant difference between the no-conflict and the no-hidden condition (*t*(26) = −1.08, *p* = 0.29).

#### Eye-Tracking Results

As neither the RTs, nor the accuracy, nor the ERP data show a difference between the conditions no-conflict and no-hidden, we restricted the analysis of the eye-tracking data to the comparison of the conflict vs. no-conflict condition.

##### Looks to target object

In our experimental setup, the identification of the correct referent (e.g., the small star) could happen only after the processing of the critical noun (1650 ms after the onset of the auditory request). In the noun time window (1650–2650 ms post auditory onset, i.e., 0–1000 ms post noun onset), the cluster-based permutation test revealed later and fewer looks to the target in the conflict condition compared to the no-conflict condition 1900–2400 ms after auditory onset (i.e., 250–750 ms after noun onset; cluster *t*-statistic: −32.37, *p* < 0.001). In the post noun time window, 2650–3650 ms post auditory onset (1000–2000 ms post noun onset), the cluster-based permutation test revealed longer looks to the target in the conflict condition compared to the no-conflict condition 3100–3650 ms after auditory onset (i.e., 1450–2000 ms after noun onset; cluster *t*-statistic: 34.00, *p* = 0.002). Thus, the integration of CG in the conflict condition delayed the looks to the target and let the participants look longer at it.

##### Looks to privileged object

**FIGURE 6:**
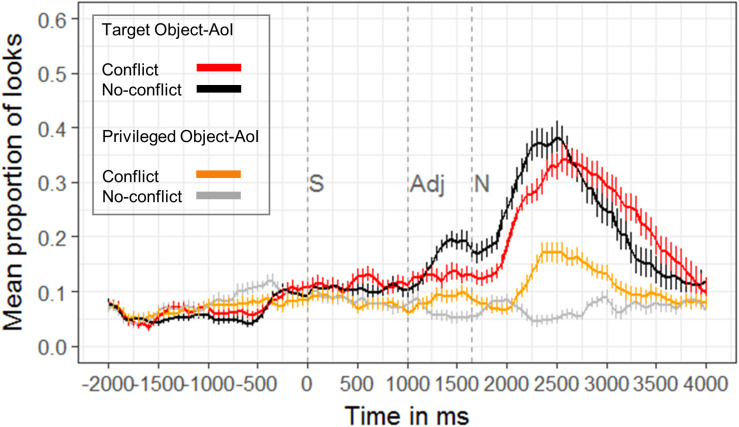
Proportion of looks to the target (red, black) in CG and to the object in privileged ground (orange, gray) in the conflict and no-conflict conditions, respectively (*n* = 27). Trials are aligned to the onset of the sentence (S), e.g., “Move the small star to the top” at 0 ms. The onset of the sentence (S, at 0 ms), the onset of the adjective (Adj, 1000 ms post onset of the auditory request), and the onset of the noun (N, 1650 ms post onset of the auditory request) are marked by dashed vertical lines in the Figure. Cluster-based permutation analyses indicated statistically significant differences between the red and the black line 1900–2400 ms and 3100–3650 ms after auditory onset and between the orange and the gray line 2100–3200 ms after auditory onset. Error bars represent the standard error (SE). For alternative Figures see Supplementary Materials.

Since looks to the target occurred later and were longer in the conflict condition, the most interesting question is, whether looks to the privileged object caused the longer latencies in the conflict condition. Cluster-based permutation analysis showed that participants indeed looked more often to the privileged object in the conflict condition as compared to the no-conflict condition from 2100 to 2650 ms post auditory onset in the noun time window (i.e., 450–1000 ms post noun onset; cluster *t*-statistic: 60.01, *p* < 0.001) and from 2650 to 3200 ms post auditory onset in the post noun time window (i.e., 1000–1550 ms post noun onset; cluster *t*-statistic: 41.10, *p* < 0.001) (Figure 6). In addition, in the Supplementary Materials we compared the looks to the privileged object in the conflict-condition to the medium sized object in the no-conflict condition (see Supplementary Figure 4). Participants looked to the privileged object in the conflict condition more often than to the medium sized object in the no-conflict condition, but still the medium-sized object might be affected by “carry-over effects” such that it was looked at, although it never was the smallest or biggest object at display.

##### Anticipatory looks to CG objects within the two object triplets

**FIGURE 7:**
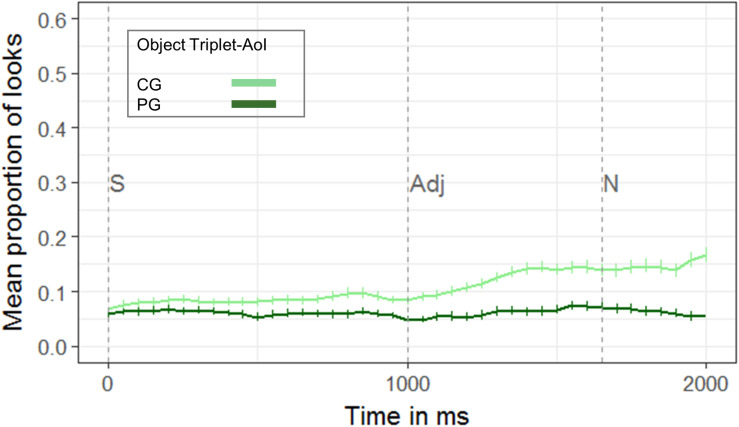
Proportion of looks to the objects in common ground (CG, light green), and privileged ground (PG, dark green), of the two object triplets in the adjective time window 1000–1650 ms post sentence onset (S) (*n* = 27). The onset of the sentence (S, at 0 ms), the onset of the adjective (Adj, 1000 ms post onset of the auditory request) and the onset of the noun (N, 1650 ms post onset of the auditory request) are marked by vertical dashed lines in the Figure. Cluster-based permutations showed that participants were more likely to look at the objects in common ground before encountering the noun (i.e., 1000–1650 ms after auditory onset). Error bars represent the standard error (SE).

Our data showed that participants anticipated objects in CG to be the referent even before the onset of the disambiguating noun (see Figure 7). The comparison using the cluster-based permutation revealed an anticipation of objects in CG in the adjective time window (cluster *t*-statistic: −50.56, *p* < 0.001). That is, 1000–1650 ms post auditory onset (i.e., right after adjective onset), participants were more likely to consider the small or big object in CG when encountering the adjective than the object in PG.

## Discussion

Our study aimed to verify different, partly conflicting accounts about when CG information is integrated during reference processing. Early integration accounts posit that CG information immediately constrains the domain in which utterances are typically processed (e.g., Nadig and Sedivy, 2002; Hanna et al., 2003; Brown-Schmidt et al., 2008; Heller et al., 2008). In contrast, late integration accounts suggest that CG information enter the parsing process at a later stage, either due to strategic egocentric processing strategies (*Egocentrism Account*, Keysar et al., 2000) or because lexical information is activated autonomously independent from perspective (*Autonomous Activation Account*, Barr, 2008). Recent accounts try to integrate both accounts and offer that perspective-taking is simultaneously affected by both the egocentric and the CG perspective (*Simultaneous Integration Account*, Heller et al., 2016).

We here combined a computerized version of the well-established referential communication game (similar to Keysar et al., 2000) with behavioral, EEG (Experiment 1), and eye-tracking (Experiment 2) measures in order to better understand the temporal dynamics and the cognitive processes underlying the integration of CG information. We analyzed three conditions: conflict, in which there was a conflict between the participant’s privileged information and CG information, and no-conflict and no-hidden, in which there was no such conflict. As neither the RTs, nor the accuracy, nor the ERP data show a difference between the conditions no-conflict and no-hidden, we can conclude that the mere presence of occluded slots in the grid did not lead to some unspecific computation of ground or did not affect general attentional processes. Therefore, our discussion will mainly focus on the comparison of the conflict vs. no-conflict condition. In the conflict condition, the object that fits the avatar’s request best (e.g., the small star) was in PG. Thus, the consideration of CG information and the suppression of PG information were necessary in order to pick the correct referent in CG. In contrast, in the no-conflict condition, the consideration of CG information was not necessary for reference resolution, because there was no conflicting information in PG to be resolved.

Comparing the conflict and the no-conflict conditions, RT data, ERP, and eye-tracking results all point to the notion that the integration of CG has its costs. This is the case even though participants initially anticipated objects in CG (the small/big item of the two object triplets, which was in CG) to be the referent until encountering the critical noun phrase, as revealed by the eye-tracking data. Then, 450–1550 ms after noun onset, eye-tracking also showed that participants were indeed distracted by privileged information in the conflict-condition: They considered the competitor although it was in PG. They also looked less to the target in the conflict vs. the no-conflict condition 250–750 ms post noun onset. Slightly later, that is 750–1250 ms post noun onset, ERPs revealed a late positivity when comparing the conflict and the no-conflict conditions. We propose that the late positivity resembled a P600-like response. Similarly to the increase in theta-band power, the P600 mirrors an increase in processing costs when discourse representations have to be monitored, updated, and modified (e.g., van Herten et al., 2005; Bornkessel-Schlesewsky and Schlesewsky, 2008; Schumacher, 2009; Burmester et al., 2014). This interpretation is also in line with the syntax-discourse model (SDM) (Burkhardt, 2005), and its extension, the multi-stream-model of discourse processing (e.g., Hung and Schumacher, 2012; Wang and Schumacher, 2013). Later on in the processing time line, in the post-noun-phase (1450–2000 ms post noun onset), eye-tracking revealed later or longer looks to the target in the conflict vs. the no-conflict condition. Finally, RTs of both the EEG and the eye-tracking cohort were significantly slower in the conflict (EEG: *M* = 1469.07 ± 364.17 ms; eye-tracking: *M* = 1570.20 ± 497.65 ms) in comparison to the no-conflict condition (EEG: *M* = 1289.38 ± 375.18 ms; eye-tracking: *M* = 1351.58 ± 526.68 ms)^1^. Overall, the RT data confirmed previous findings that RTs are longer when another person’s visual perspective is inconsistent with one’s own perspective than when it is consistent (e.g., Qureshi et al., 2010; Samson et al., 2010; McCleery et al., 2011; Wang et al., 2019) and are thus also in line with late integration accounts.

Only the accuracy results did not reveal any differences for the conflict vs. no-conflict condition. Accuracy was high for both conditions in the EEG and the eye-tracking cohort. Even in the conflict condition, participants rarely made any errors (EEG: *M* = 0.12 ± 0.42%; eye-tracking: *M* = 0.40 ± 1.14%). Only 15% of those few errors were egocentric errors. These arose in the conflict condition when participants were not able to suppress privileged information when choosing a referent. Since the accuracy data does not give rise for a strong tendency to use an egocentric strategy, the *Egocentrism Account* (Keysar et al., 2000) cannot explain our data. Given that our design was most similar to that of Keysar et al. (2000), we suppose that the clear two-step instruction, the practice phase with corrective feedback (Wang et al., 2016), and the high repetition rate eliminated possible egocentric errors.

Our eye-tracking data therefore suggest that listeners initially start with the expectation that the speaker refers to an object that is shared (i.e., is in CG). This is in line with accounts that assume early effects of CG (Nadig and Sedivy, 2002; Hanna et al., 2003; Heller et al., 2008) or early attempts to take a speaker’s perspective in anticipation of a referring expression (Barr, 2008). However, with the presentation of the noun, they also consider the mentioned objects, even if they are in PG. This information seems to interfere with the earlier tendency to consider objects in CG to be the referent. The interference of privileged information happened 450–1550 ms after noun onset. There was an increase in looks to the competitor in the conflict-condition, while looks to the target were reduced (250–750 ms after noun onset). Finally, longer looks to the target were registered in the conflict vs. no-conflict condition in the later post-noun phase (1450–2000 ms after noun onset). This interference of the privileged competitor makes the late integration of CG information an effortful process, since the current discourse model has to be updated and modified (e.g., Hung and Schumacher, 2012).

Our ERP data support this view as they point to processing differences between conditions as well. Late positivities were identified that differentiated the neuronal responses to the conflict and the no-conflict condition. First, around 750–850 ms after the onset of the critical noun we found a positivity that had a posterior distribution. Second, after around 1000 ms a positivity was observed that had a more anterior distribution. These late positivities were taken as indication for increased processing costs due to the updating of discourse representations and conflict resolution (Burkhardt, 2007; Schumacher, 2009). Especially the earlier positivity may reflect a P600-like response (for a review, see Swaab et al., 2012), since it occurred over posterior brain regions, was not lateralized, and the positive slow deflection started around 500 ms post noun onset (but became significant in the statistical analysis only after 750 ms). Although modulations of the P600 were initially attributed to syntactic anomalies or ambiguities, its functional interpretation has been extended considerably in the last decades (e.g., Brouwer et al., 2012, 2017). The P600 seems to be evoked when some kind of information needs to be integrated into the unfolding interpretation of the sentence or a reanalysis has to be undertaken due to inconsistent streams of semantic, morphosyntactic, and pragmatic information (e.g., Kuperberg et al., 2007). The same holds for executive or cognitive control mechanisms in error monitoring or information-reprocessing due to response uncertainties during language comprehension (e.g., van Herten et al., 2006). The SDM, that was first introduced for pronominal-antecedent relations by Burkhardt (2005), and extended to general discourse processing in a multi-stream-model (e.g., Hung and Schumacher, 2012; Wang and Schumacher, 2013), interprets late positivities as being induced by discourse updating and discourse modification. Finally, *P600-as-P3-accounts* (e.g., Sassenhagen et al., 2014; Sassenhagen and Fiebach, 2019) question the language-specificity of the P600 but rather see them as a (domain-general) component indexing the linkage of saliency and response selection. Our data do not allow to disentangle the underlying mechanisms, however, our results of Experiment 1 indicate that participants integrate CG information relatively late and in an effortful manner. RTs were slower for the conflict than for the no-conflict and no-hidden conditions. We interpret our ERP data as pointing to increased processing demands when CG needs to be considered (conflict condition) compared to when there is no conflict between CG and privileged information (no-conflict and no-hidden condition). As the RT data indicate that the conflict-resolution had its cost, we consider the positivity as indexing this increased processing cost. This interpretation would go in line with a domain-general view on the ERP positivities as in the *P600-as-P3-account*, in which the positivity is considered to index behaviorally relevant saliency (Sassenhagen et al., 2014).

Taken all findings together, we found very little egocentric errors, or other differences in response accuracy. Yet RTs, EEG, and eye-tracking data showed that CG integration seems to be an effortful, long-lasting, and rather late process. We saw evidence for an early anticipation of CG objects, but privileged information could not be fully neglected, resulting in a late integration of CG information during the parsing process. Therefore, our data can be explained by one of the late integration accounts, namely the *Autonomous Activation Account* of Barr (2008). This account suggests early anticipation without (or with late) integration of CG information. The *Simultaneous Integration Account* of Heller et al. (2016) posits that both egocentric and CG behavior are simultaneously active during perspective taking. Crucially, the specific design or context triggers, which behavior-egocentric or CG-dominates the task performance. As a result, the integration of CG information varies from task to task and from design to design. In our study, the privileged object best matched the referring expression, as it was the case in the Keysar et al. (2000) study. Heller et al. (2008) argue that in such cases, the “goodness of fit” to the speaker’s referring expression strongly enforces attention to the privileged object. This makes the interference with CG information more likely than in previous designs that support early integration accounts (for a thorough discussion please refer to Heller et al., 2008). Our findings thus do not speak against the *Simultaneous Integration Account* of Heller et al. (2016) but support the conclusions drawn from their work.

As outlined in the introduction, Sikos et al. (2019) used EEG to study perspective-taking using a somewhat different design than we did and interpreted their data in the framework of early integration, constrained-based accounts. Their sentences were locally ambiguous [e.g., “Click on the brontosaurus with the boots” with two brontosauri in the display, both in CG (Common Ground Competitor condition) or one in CG and one in PG (Privileged Ground Competitor condition)]. The disambiguating noun (e.g., “boots”) was always presented at the end of the sentence in both competitor conditions. In addition, there was a No-Competitor Control condition with just one brontosaurus in CG and a perceptual control condition. The PG Competitor condition in Sikos et al. (2019) is temporally (i.e., until the onset of the disambiguating noun) similar to our conflict condition. The other conditions can’t be directly “translated” to our design: both the No-Competitor Control condition and the CG Competitor condition are similar to our no-conflict condition; in the No-Competitor condition the utterance is already disambiguated at the first noun (“brontosaurus”) while in the CG Competitor condition, the disambiguation comes later (“with the boots”). Similar to our study, Sikos et al. (2019) find increased RTs in both competitor conditions (PG Competitor, CG Competitor) as we find them in our conflict condition. However, in the ERP data they find a late, widely distributed, negativity for the CG Competitor condition. The authors interpret this as an Nref component (van Berkum et al., 1999, 2003), indicating that only the CG competitor is considered as a potential referential candidate, but not the competitor in PG. Nieuwland et al. (2007) take the frontal negative shift as reflecting genuine referential ambiguity in the current model of the discourse in a deeper sense, which is related to referential accessibility. Looking at the ERP patterns in Figure 3 of Sikos et al. (2019), (page 281) it looks as if the PG Competitor condition (i.e., the conflict condition in our terminology) leads to a greater positivity than the CG Competitor condition. In our data we interpret this as a late positivity for the conflict condition indexing updating costs. However, in Sikos et al. (2019) this difference seems not to be statistically significant and not different from the No-Competitor condition. If we apply the Nref-argument to our data and interpret our data as an Nref component, the no-conflict condition would show the Nref effect compared to the conflict condition (i.e., more negative in no-conflict than in conflict trials). However, this seems unlikely, in our opinion, as in our no-conflict condition there is clearly only ONE potential referent (e.g., the small star) which is in CG. This would contradict the assumption that the Nref indexes the accessibility of MULTIPLE potential referents as in Sikos et al. (2019). This interpretation would only apply, if the medium-sized star is not considered as “medium” but as a second small or second big star at the display leading to referential ambiguity. We cannot completely exclude this possibility. An additional analysis of looks to the medium-sized object reveals, that participants also look at this object (see Supplementary Material). This indicates that participants’ perspective regarding what constitutes a “small” or “big” object might be shifted, and that considering the medium-sized object as a good referent for “small” or “big” is carried over to conditions where perspective-taking is not necessary (i.e., the no-conflict condition). But, firstly, looks to the target by far exceed looks to the medium-sized object. Secondly, the supplementary analysis reveals that participants looked more often to the privileged object (i.e., the competitor that is either the small or big object) in the conflict condition as compared to medium-sized object in CG in the no-conflict condition. Thirdly, accuracy shows that participants almost always choose the correct object, which is the smallest or biggest star at the display in the no-conflict and no-hidden condition. This indicates that our participants indeed considered the medium-sized star as medium, otherwise the potential referential ambiguity would not have been disambiguated and a referential choice could not have been made. Overall, while our study design differs from Sikos et al. (2019), we find similar behavioral effects and ERP-patterns which, superficially, look similar but which are interpreted differently. Sikos et al. (2019) conclude that the competitor in PG is not considered a potential referential candidate and that the RT effects just reflect attentional distraction effects. The latter effects are, however, not mirrored in their ERP data as Sikos et al. found no difference between the PG Competitor and the No-Competitor condition. Accuracy rates were very high in all conditions in Sikos et al. (2019) which the authors interpret as indicating that competitors in PG are not considered a potential candidate. However, if this would be generalized to all kinds of perspective taking tasks, “egocentric errors,” as have been shown in Keysar et al. (2000), or the finding of interference from information in PG (e.g., Barr, 2008) would be hard to explain. Also, if we consider our own eye-tracking data, the assumption, that PG information is not considered as a potential referent, becomes implausible: The eye-tracking data show, that the longer RTs in the conflict condition in comparison to the no-conflict condition can be attributed to two interfering effects. The first interfering effect resulted from a strong anticipation of an object in CG to be the referent: when encountering the adjective, participants showed higher proportions of looks to the object in CG compared to the object in PG in both object triplets. Then, the second interfering effect arose. When encountering the noun, participants shifted their attention to the object of comparison in PG in the conflict condition (i.e., they shifted their attention to the “egocentric competitor”): 450–1550 ms after noun onset, there was an increase in looks to the object of comparison in PG in the conflict condition (i.e., to the competitor). Only later, 1450–2000 ms after noun onset, looks to the competitor decreased while looks to the target (in CG) were reaching their peak.

To conclude, our data speak in favor of the *Autonomous Activation Account* (Barr, 2008), since our eye-tracking data reveal an early anticipation (in the adjective time window) but a late, effortful integration of CG information (in the noun time window). The re-evaluation or integration seems to be a late and effortful process reflected by increased processing costs (RTs), later and longer looks to the target, and late positive and slow brain responses. However, the data can also be aligned to the *Simultaneous Integration Account* of Heller et al. (2016), since, overall, listeners restrict their referential domain to information in CG when appropriate, but the information in PG has the potential to interfere. The *Simultaneous Integration Account* elegantly combines the contrary findings of egocentric vs. CG behavior and early vs. late integration of CG that can be found in the literature. Further, it highlights the circumstances of such performance differences. Yet, how fast CG information affects reference processing seems to depend on a variety of factors such as the current communicative and experimental setting, the familiarity to the interlocutor (e.g., Münster and Knoeferle, 2017), the complexity of task demands, or just the readiness or motivation to take another person’s perspective. The establishment of a model of socially situated language processing, which incorporates all these factors, should be further addressed in the future.

## Data Availability Statement

The datasets generated for this study can be found on the Open Science Framework, https://osf.io/vdsxu/.

## Ethics Statement

The studies involving human participants were reviewed and approved by Ethics Committee of the University of Potsdam. The patients/participants provided their written informed consent to participate in this study.

## Author Contributions

MR was responsible for designing and implementing the experimental paradigms and for collecting and analyzing the data and wrote the manuscript. MP was involved in data collection and data analysis and was involved in writing the manuscript. IW and BH designed the study and contributed to writing and revising the manuscript. All authors contributed to the article and approved the submitted version.

## Conflict of Interest

The authors declare that the research was conducted in the absence of any commercial or financial relationships that could be construed as a potential conflict of interest.

## Footnotes

1 It is not quite clear why participants responded around 100 ms slower in the eye-tracking cohort than in the EEG cohort. One possibility might be that participants were more aware of being monitored by the eye-tracker than the EEG and hence showed a more careful responding behavior (click, drag-and-drop). In addition, as outlined above, in Experiment 2 we prevented that the target and the privileged object in the grid appeared in neighboring slots of the grid to minimize the misclassification of looks. This also might account for slightly longer reaction times. However, the differences between the means of the conflict and the no-conflict conditions were similar in both groups (ERPs: 179.69 ms, eye-tracking: 218.62 ms), and the statistical analyses pointed to the same direction.
