# Supplementary material for: Common Ground Information Affects Reference Resolution: Evidence From Behavioral Data, ERPs, and Eye-Tracking
Source: Front Psychol. 2020 Nov 30;11:565651. doi: 10.3389/fpsyg.2020.565651 (PMC7734025; doi:10.3389/fpsyg.2020.565651)
Supplement: Supplementary file 1 [file Data_Sheet_1.PDF]

Supplementary Materials

A Time-frequency analysis of EEG data

1 Methods

The calculation of power at different frequencies over a time interval allows for the analysis of time and frequency information at once (time-frequency analysis, TFA). Oscillations are thought to be a basic form of communication between neurons. The coupling and uncoupling of functional networks in the brain can be related to patterns of neural synchronization and desynchronization. TFA is therefore of special interest for cognitive research, since neurons in the same functional network oscillate synchronously at a given frequency. The frequency bands that are commonly examined in cognitive research are the theta band (4-7 Hz), alpha band (8-12 Hz), beta band (12-30 Hz), and gamma band (30-100 Hz). The manipulation of a certain experimental stimulus induces amplitude increases and decreases in the specific frequency bands, and may provide a link to brain functions (for a review in language comprehension see Meyer, 2017). For studies of pragmatics in particular, TFA was shown to add information beyond what ERPs could reveal. In particular, Hagoort and colleagues (2004) investigated whether sentences containing world knowledge violations are processed differently than sentences containing semantic violations. Using the N400 ERP component, they could not detect significant differences between these two types of violations; however, a TFA revealed significant differences between world knowledge violations and semantic violations in the gamma band, related to feature binding, and the theta band, related to episodic or working memory (Hagoort et al., 2004). Similarly, van den Brink and colleagues (2012) demonstrated that the integration of semantic and social information in linguistic utterances can be linked to distinct frequency bands. In their study, semantic violations were related to an increase in the theta band, whereas speaker identity violations were related to the gamma band. We here explore whether differences in CG processing in the time frequency domain will provide further information about the underlying brain functions.

1.1 Participants

Please refer to section "Participants" of Exp. 1 in the main manuscript for general participant different preprocessing steps (please refer to paragraph 1.5 of the supplemental material), 30 information. Due to participants entered the final TFA analysis.

1.2 Materials and Design

The materials and the design are outlined in section "Materials and Design" of Exp. 1 in the main manuscript.

1.3 Procedure

Section "Procedure" of Exp. 1 of the main manuscript describes the procedure of the experimental setup.

1.4 EEG recordings

Section "EEG Recordings" of Exp. 1 of the main manuscript describes the details of the EEG recordings.

1.5 Time-frequency analysis

The TFA analysis requires different preprocessing steps and settings (e.g., length of baseline, length of segments, filter settings) than ERP data analysis. The data preprocessing here was performed with the Fieldtrip toolbox for EEG/MEG analysis (Oostenveld et al., 2011) in MATLAB® (2015b, MathWorks, Natick, MA).

As a first step, the raw EEG-signal was re-referenced offline to the linked mastoids. A Hamming-windowed 3rd-order 0.01 Hz Butterworth high-pass filter and a 100 Hz low-pass filter of the same type were applied. In order to remove line noise, a 50 Hz notch filter was used. The signal was then segmented into trials beginning at 1700 ms before the onset of the noun, and ending at 2500 ms after noun onset. This relatively wide time window was chosen to avoid boundary effects in the time segments of interest. Then, a semi-automatic artifact rejection procedure was applied to reject muscle and jump artifacts (spikes) on a trial-by-channel basis. For the muscle artifacts, the segments were band-pass-filtered at 110 to 140 Hz. For both muscle and jump artifacts, the signal was z-transformed to obtain a z-distribution of all observed trial amplitudes. The cut-off z-value for muscle artifacts was set to 7, for jump artifacts to 20. The data was finally screened manually on a trial by channel basis to remove further artifacts. Moreover, trials in which incorrect responses occurred were removed. Only participants with a minimum of 40 trials per condition entered the final analysis (i.e., n=30).

To remove ocular artifacts, an independent component analysis (ICA) was performed. Importantly, ICA is a method that corrects artifacts instead of removing them. The method is based on the assumption that each type of artifact as well as brain component has a certain topography and signature. An ICA decomposes the EEG signal into a number of independent components that correspond to the degrees of freedom (i.e., the number of electrodes minus one). The resulting components were visually inspected. Based on their topographies and the time course, components related to ocular artifacts, that is, blinks and eye movements, were identified. Using backpropagation, the components classified as ocular artifacts were subtracted from the data.

The TFA was performed in 50 ms steps from -1700 to 2500 ms in 2 Hz steps from 1 to 29 Hz using Hanning tapers with a time window length of 600 ms. For 30 to 100 Hz, we used multitapers with the same time window length. Simultaneously, the data were also demeaned and detrended. Then, averaging was performed across all participants to obtain grand averaged time-frequency data.

For statistical analysis, cluster-based permutation tests were performed (Maris & Oostenveld, 2007) in order to deal with the multiple comparisons problem. The statistics was run two-tailed and within-subjects, with a minimum number of two significant ($\alpha<0.05$) electrodes to form a cluster. 50 ms running time windows were calculated. We considered clusters as significant when they were significant over the entire 50 ms time window. Data were randomly permutated 1000 times.

2 Results

The cluster-based permutation analysis revealed a significant positive channel-time cluster for the comparison of the **conflict vs. no-conflict** conditions in the theta-band (3-7 Hz; cluster-*t*: 1121; p=0.003) in the 400-1100 ms time window post noun onset (see Supplementary Figure S1).

The comparison between the **conflict and the no-hidden** conditions also showed significant positive channel-time clusters in the theta-band 400-1400 ms post noun onset (cluster-*t*: 1443; p=0.001) (see

Supplementary Figure S2) and in addition in the gamma-band, namely 400-800 ms (34-100 Hz, cluster-t = 4691, p = 0.011) and 1300-1700 ms post noun onset (36-100 Hz, cluster-t = 8456, p = 0.003; see Supplementary Figure S3). Moreover, there were negative channel-time clusters, that is desynchronizations, in the alpha-band and in the beta-band 1750-2150 ms post noun onset (9-23 Hz, cluster-t=1200, p=0.001). These results showed that finding the correct referent in a scenario which afforded to make use of CG information (i.e., conflict condition) in contrast to a scenario in which no CG information had to be considered (i.e., no-hidden and no-conflict conditions) led to power increases in the theta and/or gamma band and/or to power decreases in the alpha- and beta-bands. All effects were in sum widespread across the entire time window and not limited to certain electrode clusters in specific brain regions.
The comparison between the **no-conflict and the no-hidden** conditions revealed no significant differences, mirroring the ERP results (see main text).

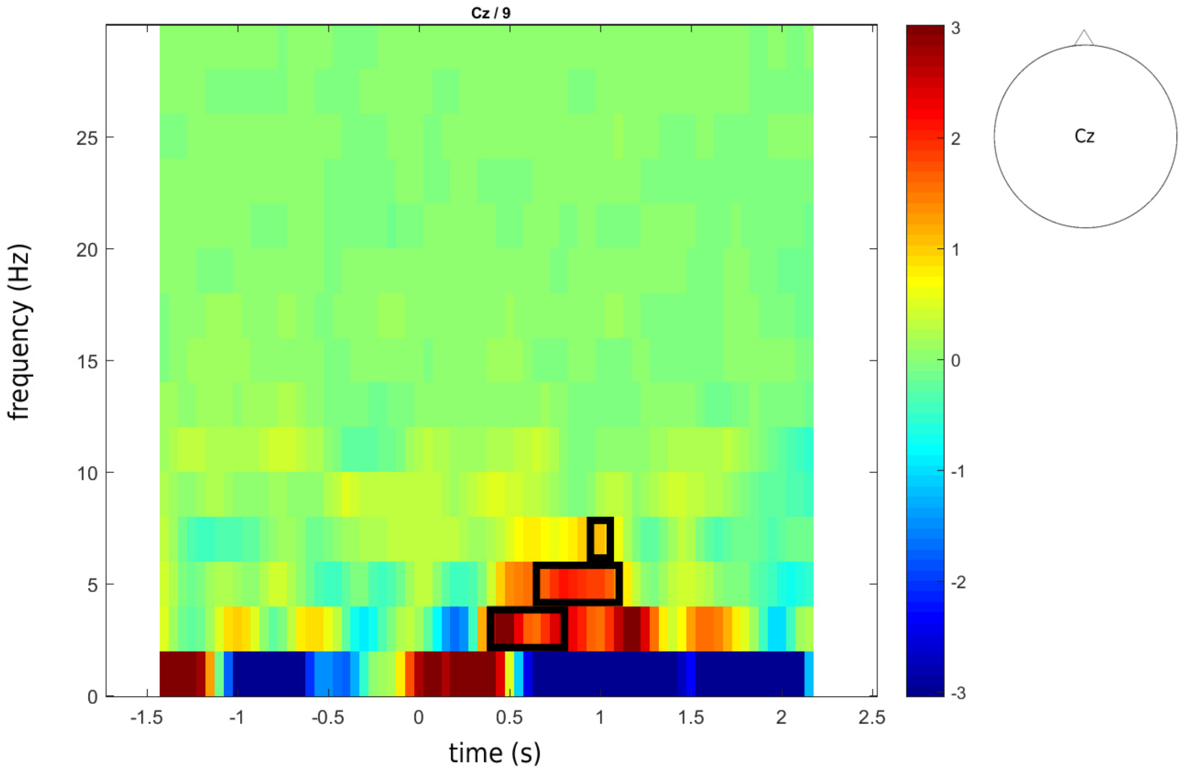

Supplementary Figure S1: Grand-averaged TFA data of conflict vs. no-conflict 1-29 Hz at the representative electrode Cz (n=30). Synchronization is indicated by yellow and red, desynchronization is indicated by light blue and dark blue. Significant positive channel-time clusters are indicated by black squares. There were no significant negative channel-time clusters. Time is expressed relative to noun onset.

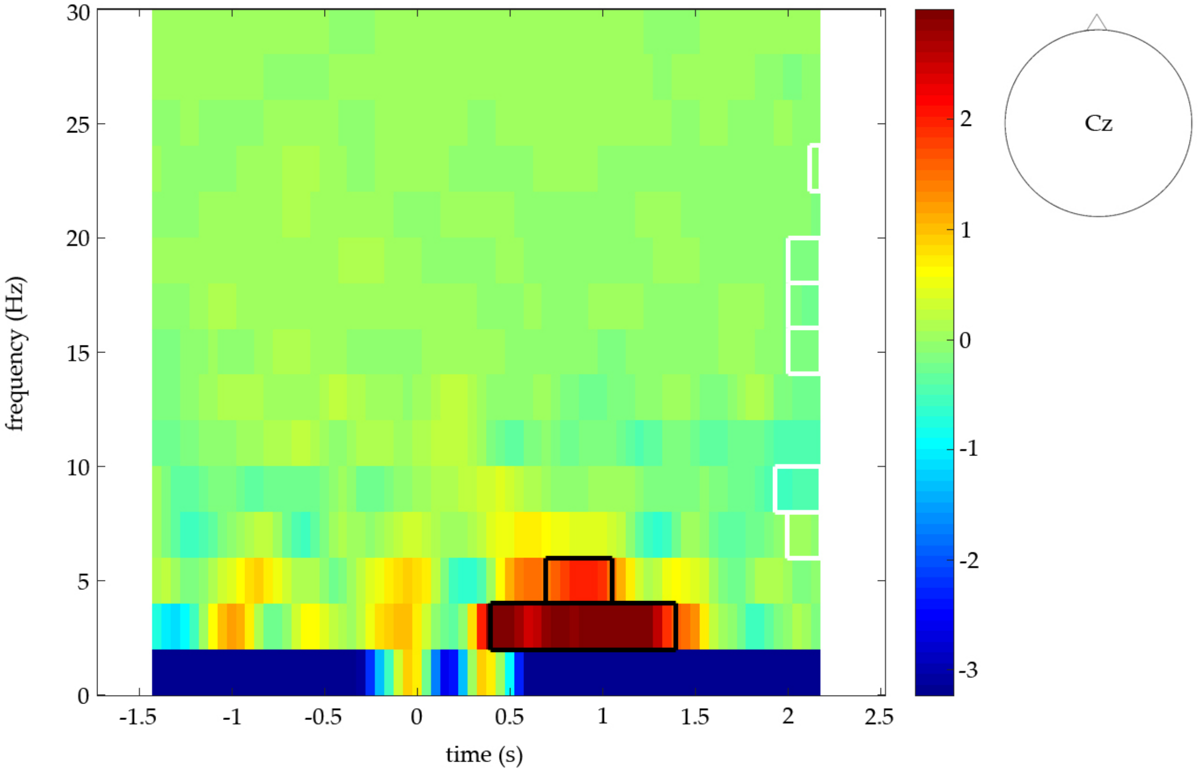

Supplementary Figure S2: Grand-averaged TFA data of conflict vs. no-hidden 1-29 Hz at the representative electrode Cz (n=30). Synchronization is indicated by yellow and red; desynchronization is indicated by light blue and dark blue. Significant positive channel-time clusters are indicated by black squares, significant negative channel-time clusters by white squares. Time is expressed relative to noun onset.

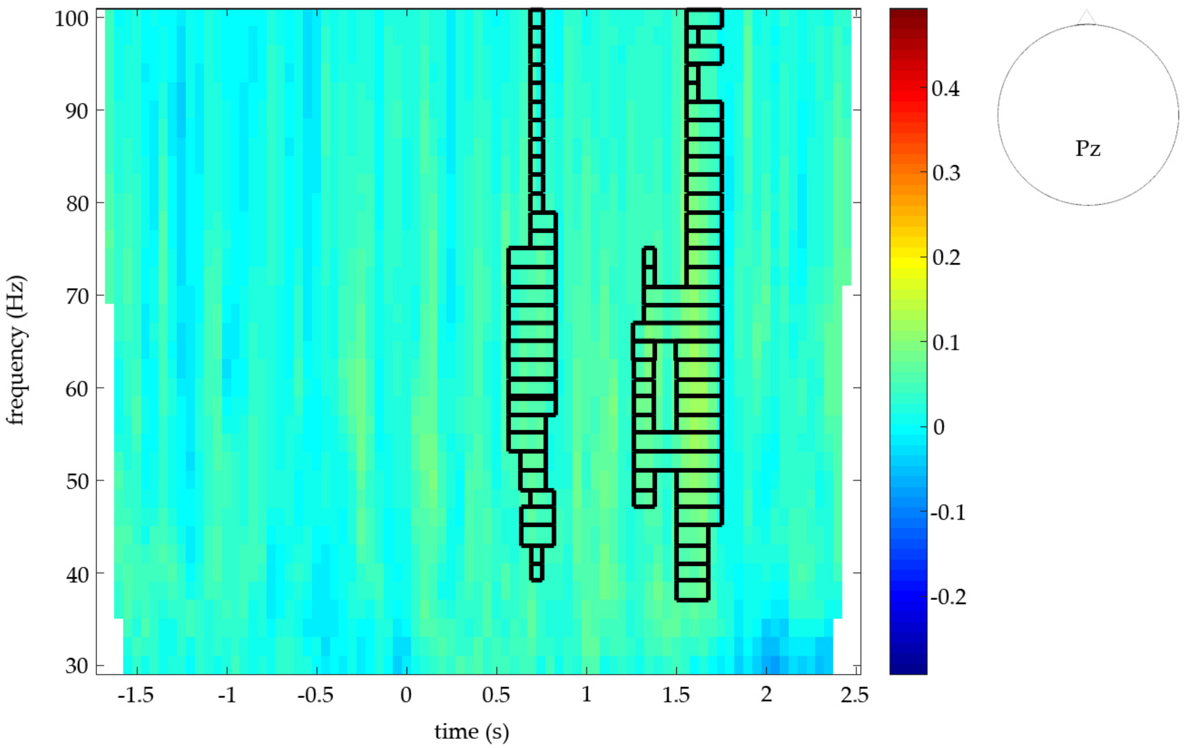

Supplementary Figure S3: Grand-averaged TFA data of conflict vs. no-hidden 30-100 Hz at the representative electrode Pz (n=30). Synchronization is indicated by yellow and red; desynchronization is indicated by light blue and dark blue. Significant positive channel-time clusters are indicated by black squares. There were no significant negative channel-time clusters. Time is expressed relative to noun onset.

3 Discussion

Time-frequency analyses have the potential to uncover how neural oscillations subserve higher-level linguistic processing. Our data showed that the integration of common ground (CG) information, which was necessary in the conflict condition, leads to power increases in the theta-band when comparing the conflict to the no-conflict condition and the conflict to the no-hidden condition. The power increase in the theta-band was widespread, not limited to a certain region. In general, increases in theta-band power are linked to syntactic tasks rather than to semantic tasks (Röhm et al., 2001; Bastiaansen et al., 2002). Moreover, there is good evidence that an event-related increase in theta band power reflects long-term memory retrieval. Bastiaansen et al. (2002), for instance, showed that theta band increases indexed the establishment of an episodic memory trace when a sentence unfolds word by word. Theta-band power increases were also consistently found under increasing working memory demands (Gevins et al., 1997; Klimesch, 1999), in language comprehension (Weiss et al., 2000; Weiss et al., 2005), as well as generally in cortical information processing (see Meyer, 2017, for a discussion). In this vein it is possible that integrating CG information in the conflict condition put increased processing demands on the participants, thus increasing working memory load or general processing load. Most interestingly for the purposes of our study, Herrmann et al. (2005) pointed out that theta oscillations can represent slow potentials in ERPs, such as P300, N400, and P600. The increase in theta-band power starting 400ms post noun onset in our study therefore most likely resembles the late positivity of the ERP component which was interpreted in a similar way (see main text).

Besides the theta-band increase, a decrease in alpha-band and beta-band power comparing the conflict vs. no-hidden conditions was visible in a late time window (1750-2150 ms post noun onset). Alpha-band oscillations have been associated with cortical inhibition (Klimesch et al., 2000). Inhibition is closely linked to the two fundamental functions of suppression and selection which allow for an orientation in time, space, and context. The release of inhibition is associated with an alpha-band power decrease. The alpha-band power decrease in our study roughly corresponds to when participants' looks to the egocentric competitor decreased and looks to the target in CG increased (1450 – 2000 ms; see section "Eye-Tracking Results" in the main text). Therefore, the alpha-band power decrease may signal the release of inhibitory mechanisms in the conflict condition that had been necessary before in order to suppress the competitor in privileged ground in favor of the target in CG.

Beta-band power is strongly associated with semantics in language comprehension, especially when lexical semantic predictions are violated. For instance, a decrease in beta-band power, or modulations of beta-band coherence were observed when upcoming words did not match the predicted word (Haarmann et al., 2002), or when sentences had to be processed that were semantically incoherent with a given discourse (Lewis et al., 2017). In this vein, it is not surprising that beta-band oscillations correlate with the amplitude of the N400, an electrophysiological component that signals the semantic "goodness of fit" (Lewis et al., 2017). Besides semantic prediction, it has been proposed that beta-band oscillations subserve top-down predictive functions across various processing levels beyond higher-level language comprehension (see Meyer, 2017 for a review). Since the decrease in beta-band power occurred late, that is, after the selection of the target, it is unlikely that it reflects the mismatch between the prediction given by the discourse and the incoming noun phrase elicited in the conflict condition. However, beta-band oscillations are also frequently reported in the motor domain (Engel & Fries, 2010). For instance, decreases in the beta-band power are associated with motor action (Başar et al., 2001), rebounding one second after the end of the motor response (Herrmann et al., 2005). Since the conflict and the no-hidden condition should not differ with respect to the motor response per se, the differences in RTs and therefore the difference in the onset of the motor response (later in the conflict than in the no-hidden condition) may evoke the decrease in beta-band power for the comparison of the conflict vs. no-hidden condition.

In addition, beta-band power oscillations have been linked to gamma-band oscillations in the literature forming a beta-gamma interplay (Lewis & Bastiaansen, 2015; Lewis et al., 2016). That is, gamma-band oscillations are believed to reflect the checking mechanism ("diagnosis of fit", Meyer, 2017, p.8) of incoming bottom-up lexical-semantic representations against the top-down contextual predictions generated by beta-band oscillations. Gamma-band activity refers to both synchrony related to pre-activated lexical representations that match the input and suppression of competing lexical representations (Lewis & Bastiaansen, 2015). Both gamma-band power decreases and increases were found when the actual semantics of the incoming words do not match the preceding context or strongly fulfill predictions respectively (for a review, see Meyer, 2017). Based on this, we cannot account for the finding of a gamma-band power increase in our study since the incoming noun in the conflict condition does not meet the contextual predictions in a way it does in the no-hidden condition, and therefore a gamma-band decrease should be expected. Alternatively, the gamma-band power increase may just broadly index social processes, as reported by van den Brink et al. (2012), that are required during perspective taking in the conflict condition but not in the no-hidden condition.

In conclusion, the exact relationship between power increases/decreases as well as synchronization and desynchronization between functional networks is still to be established in the future. Our findings and interpretations therefore have a descriptive character and our analyses should be considered as highly explorative in nature.

B Additional Figures presenting looks to the medium-sized object-AOI (eye-tracking)

As an alternative look at the eye-tracking data we here illustrate the looks to the medium-sized object-AoI. In the conflict condition, the medium-sized object represents the target. In the no-conflict condition, the medium-sized object represents the "second-best" object. Figure S4 depicts how looks to the medium-sized object-AoI unfolded in the conflict and no-conflict conditions.

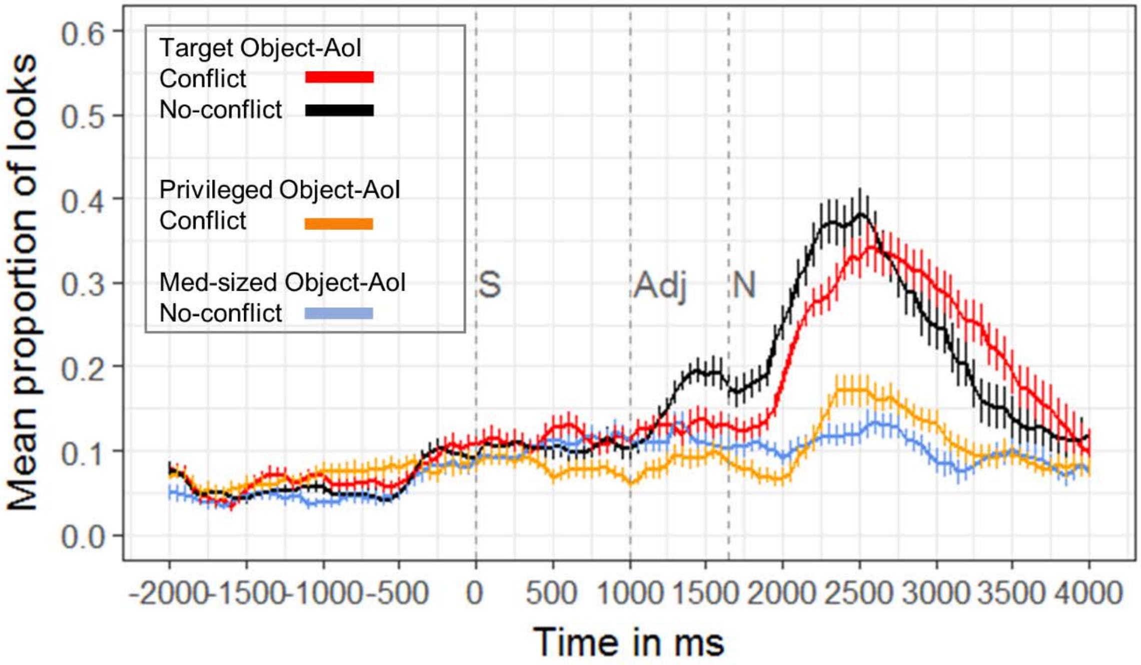

Supplementary Figure S4: Proportion of looks to the target (red, black) in CG in the conflict- and no-conflict condition, respectively. Proportion of looks to the medium-sized object in CG in the no-conflict condition (light blue) and to the object in PG in the conflict condition (competitor, orange) (n=27). Trials are aligned to the onset of the sentence (S), e.g., "Move the small star to the top" at 0 ms. The onset of the sentence (S, at 0 ms), the onset of the adjective (Adj, 1000 ms post onset of the auditory request), and the onset of the noun (N, 1650 ms post onset of the auditory request) are marked by dashed vertical lines in the Figure. Error bars represent the standard errors (SE)

We analyzed the difference between the PG competitor object in the conflict condition (e.g., small star; orange line in Figure S4) to the medium sized CG object in the no-conflict condition (blue line in Figure S4).

Results: A cluster-based permutation analysis showed that participants looked more often to the privileged object (i.e., the competitor that is either the small or big object, orange line in Figure S4) in the conflict condition as compared to medium-sized object in CG in the no-conflict condition (blue line in Figure S4) from 2850-3050 ms post auditory onset (i.e., 1200-1400 ms post noun onset; cluster

t-statistic: 10.22, *p*=0.034). Earlier in the noun time window, cluster-based permutation analysis revealed a trend that participants looked more often to the privileged object in the conflict condition as compared to medium-sized object in the no-conflict condition from 2350-2500 ms post auditory onset (i.e., 700-850 ms post noun onset; cluster *t*-statistic: 8.33, *p*=0.057). We interpret these looks to the privileged competitor as indicating that participants might not to be able to fully suppress their privileged, egocentric perspective, when confronted with conflicting information. But still, the medium-sized object seems to be affected by "carry-over effects" (blue line in Figure S4) such that it was looked at, although it never was the smallest or biggest object at display. This indicates that participants' perspective regarding what constitutes a "small" or "big" object might be shifted, and that considering the medium-sized object as a good referent for "small" or "big" is carried over to conditions where perspective-taking is not necessary.

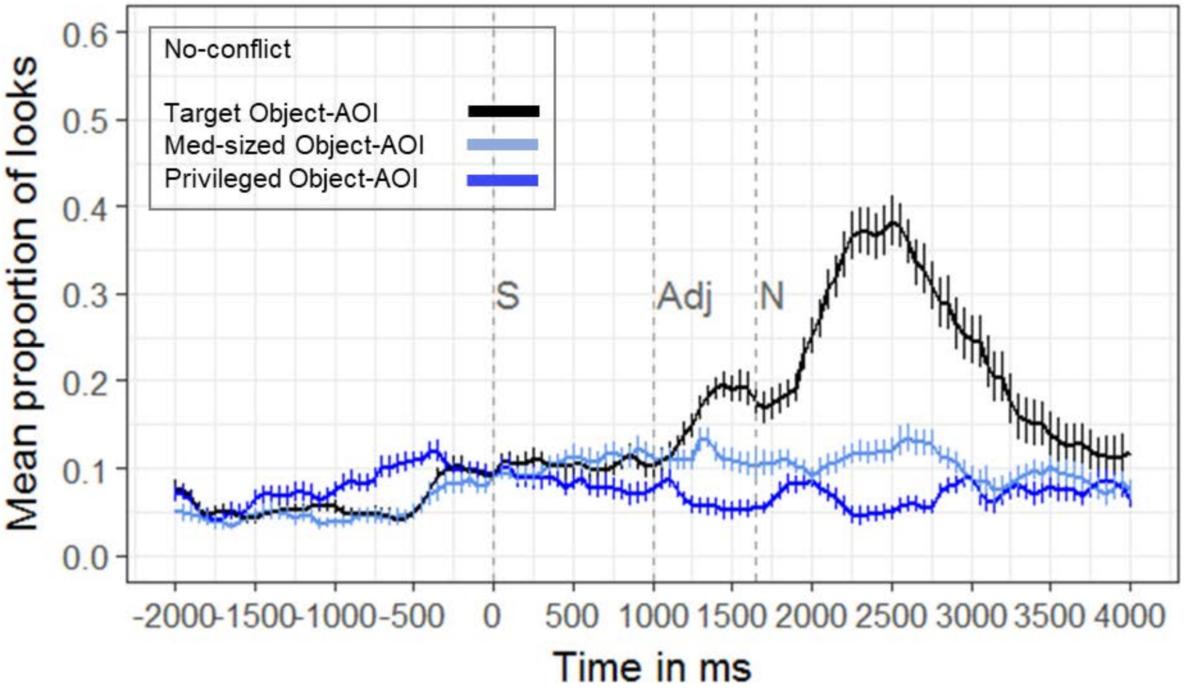

Supplementary Figure S5: Proportion of looks to target object-AoI (black), the medium-sized object-AoI (light blue), and the privileged object-AoI (blue) in the no-conflict condition (n=27). Trials are aligned to the onset of the sentence (S), e.g., "Move the small star to the top" at 0 ms. The onset of the sentence (S, at 0 ms), the onset of the adjective (Adj, 1000 ms post onset of the auditory request), and the onset of the noun (N, 1650 ms post onset of the auditory request) are marked by dashed vertical lines in the Figure. Error bars represent the standard errors (SE).

Figure S5 reveals that after hearing "small" or "big" participants did not strictly focus on the smallest or biggest object at display (black). Instead they also seem to consider the medium-sized object (light blue) as the requested "small" or "big" target object (black). Yet they considered medium-sized object to a much lesser extent than the smallest or biggest object. Moreover, they did not select the medium-

sized object as referent, as confirmed by the accuracy data. They also looked far more often to the target, the smallest or biggest object at display (black).
In this vein, it might be possible that our participants could not remember the absolute size of the objects when only looking at one of them – that is, the judgment of the size of the object might be relative and therefore looks to the other objects were elicited to compare their relative sizes. Note however, that there was a preview phase of 500 ms before the auditory instruction started. In this preview phase the relative size difference could have been evaluated. We cannot exclude that our design actually triggered the consideration of the medium-sized object in the no-conflict condition, because the medium-sized object was the target in the conflict-condition. However, if we directly compare the medium-sized objects in the conflict- and no-conflict condition (i.e., red vs. light blue in Figure S4), more looks in the conflict-condition (red) are clearly evident.
Despite all the limiting factors of our design, it seems that the participants look much more often to the competitor in PG in the conflict-condition and we take this as evidence that the PG object is interfering and is indeed considered a true potential candidate for reference.

References

Başar, E., et al. (2001). Gamma, alpha, delta, and theta oscillations govern cognitive processes. *International Journal of Psychophysiology*, *39*, 241–248. DOI: 10.1016/S0167-8760(00)00145-8.

Bastiaansen, M. C.M., van Berkum, J. J.A., & Hagoort, P. (2002). Event-related theta power increases in the human EEG during online sentence processing. *Neuroscience Letters*, *323*, 13–16. DOI: 10.1016/S0304-3940(01)02535-6.

Engel, A. K., & Fries, P. (2010). Beta-band oscillations--signalling the status quo? *Current opinion in neurobiology*, *20*, 156–165. DOI: 10.1016/j.conb.2010.02.015.

Gevins, A., et al. (1997). High-resolution EEG mapping of cortical activation related to working memory: effects of task difficulty, type of processing, and practice. *Cerebral Cortex*, *7*, 374–385.

Haarmann, H. J., Cameron, K. A., & Ruchkin, D. S. (2002). Neural synchronization mediates on-line sentence processing: EEG coherence evidence from filler-gap constructions. *Psychophysiology*, *39*, 820–825.

Hagoort, P., et al. (2004). Integration of word meaning and world knowledge in language comprehension. *Science*, *304*, 438–441.

Herrmann, C. S., Grigutsch, M., & Busch, N. A. (2005). EEG oscillations and wavelet analysis. In T. C. Handy (Ed.), *Event-Related Potentials: A Methods Handbook*. Cambridge, Massachusetts: MIT Press.

Klimesch, W. (1999). EEG alpha and theta oscillations reflect cognitive and memory performance: a review and analysis. *Brain Research Reviews*, *29*, 169–195. DOI: 10.1016/S1364-6613(99)01311-X.

Klimesch, W., et al. (2000). Simultaneous desynchronization and synchronization of different alpha responses in the human electroencephalograph: a neglected paradox? *Neuroscience Letters*, *284*, 97–100.

Lewis, A. G., et al. (2016). A Predictive Coding Perspective on Beta Oscillations during Sentence-Level Language Comprehension. *Frontiers in human neuroscience*, *10*, 85. DOI: 10.3389/fnhum.2016.00085.

Lewis, A. G., et al. (2017). Discourse-level semantic coherence influences beta oscillatory dynamics and the N400 during sentence comprehension. *Language, Cognition and Neuroscience*, *32*, 601–617. DOI: 10.1080/23273798.2016.1211300.

Lewis, A. G., & Bastiaansen, M. (2015). A predictive coding framework for rapid neural dynamics during sentence-level language comprehension. *Cortex*, *68*, 155–168. DOI: 10.1016/j.cortex.2015.02.014.

Maris, E., & Oostenveld, R. (2007). Nonparametric statistical testing of EEG- and MEG-data. *Journal of Neuroscience Methods*, *164*, 177–190. DOI: 10.1016/j.jneumeth.2007.03.024.

Meyer, L. (2017). The neural oscillations of speech processing and language comprehension: state of the art and emerging mechanisms. *The European journal of neuroscience*. DOI: 10.1111/ejn.13748.

Oostenveld, R., et al. (2011). FieldTrip: open source software for advanced analysis of MEG, EEG, and invasive electrophysiological data. *Computation Intelligence and Neuroscience*.

Röhm, D., et al. (2001). The role of theta and alpha oscillations for language comprehension in the human electroencephalogram. *Neuroscience Letters*, *310*, 137–140.

van den Brink, D., et al. (2012). Empathy matters: ERP evidence for inter-individual differences in social language processing. *Social cognitive and affective neuroscience*, *7*, 173–183. DOI: 10.1093/scan/nsq094.

Weiss, S., et al. (2005). Increased neuronal communication accompanying sentence comprehension. *International journal of psychophysiology official journal of the International Organization of Psychophysiology*, *57*, 129–141. DOI: 10.1016/j.ijpsycho.2005.03.013.

Weiss, S., Müller, H. M., & Rappelsberger, P. (2000). Theta synchronization predicts efficient memory encoding of concrete and abstract nouns. *NeuroReport*, *11*, 2357–2361.
